# Acute kidney injury: pathogenesis and therapeutic interventions

**DOI:** 10.1186/s43556-025-00293-4

**Published:** 2025-09-05

**Authors:** Xiaoqin Xu, Tingting Zeng, Si Chen, Na Tian, Chunying Zhang, Yuemei Chen, Shanying Deng, Zhigang Mao, Juan Liao, Tonghao Zhang, Yi He, Wei Wang, Pan Chen, Yali Song

**Affiliations:** 1 Department of Laboratory Medicine, Clinical Laboratory Medicine Research Center, West China Hospital, Sichuan University, Sichuan Clinical Research Center for Laboratory Medicine, Chengdu, Sichuan Province 610041 People’s Republic of China; 2 Department of Clinical Laboratory, Shanxi Province Cancer Hospital, Shanxi Hospital Affiliated to Cancer Hospital, Chinese Academy of Medical Sciences, Cancer Hospital Affiliated to Shanxi Medical University, Taiyuan, Shanxi Province 030013 People’s Republic of China; 3 Department of Anesthesiology, Qingdao Eighth People’s Hospital, Qingdao, Shandong Province 266000 People’s Republic of China; 4 Department of Statistics, University of Virginia, Charlottesville, 22903 USA; 5 Gastroenterology and Urology Department II, Hunan Cancer Hospital/the Affiliated Cancer Hospital of Xiangya School of Medicine, Central South University, Clinical Research Center for Gastrointestinal Cancer in Hunan Province, Changsha, Hunan Province 410013 People’s Republic of China; 6 Hunan Cancer Hospital and the Affiliated Cancer Hospital of Xiangya School of Medicine, Central South University, Changsha, Hunan Province 410013 People’s Republic of China

**Keywords:** Acute kidney injury, Pathophysiology, Risk, Diagnosis, Therapy

## Abstract

Acute kidney injury (AKI) is a prevalent clinical condition that is associated with unacceptably high morbidity and mortality, as well as the development of chronic kidney disease (CKD). The pathogenesis of AKI is highly complex and heterogeneous, primarily attributed to metabolic disturbances arising from the disease itself and the administration of medications related to treatment. In recent years, AKI in cancer patients is highly concerned. The emergence of AKI caused injuries and dysfunction of remote organs but also enhanced the health-care costs. It's essential for early recognition of AKI by biomarker or prediction models and further, take a timely intervention. This review aims to provide the pathophysiology of AKI covering the intricate mechanisms underpinning AKI in the dynamic context of the clinical setting, the tailored role of inflammation and ischemia, and the cellular and molecular crosstalk pathways involved. These events closely related to patients at high risk of AKI and underscore the characteristics that may make these patients more susceptible to injury. Furthermore, the diagnosis of AKI relies on clinical criteria, biomarkers, and imaging, but it should be distinguished from CKD. Finally, the review offers the therapeutic intervention in clinical practice and preclinical or clinical trials, focusing on the improvement of conventional therapy and advanced novel treatment strategies. Simultaneously, the challenge and future direction on early identifying renal impairment and performing renoprotection are also discussed, further supporting the novel discipline including onco-nephrology. The development of effective interventions that reduce nephrotoxicity is highly contingent upon a thorough understanding of the molecular pathophysiology of AKI.

**Supplementary Information:**

The online version contains supplementary material available at 10.1186/s43556-025-00293-4.

## Introduction

Acute kidney injury (AKI), a significant global health concern, is marked by a swift deterioration in renal function, leading to high rates of mortality and morbidity. The incidence of AKI is on the rise, with causes ranging from trauma, sepsis, and surgery to nephrotoxic drugs, ischemia–reperfusion injury (IRI), and cirrhosis [1, 2]. Estimates of AKI prevalence range from < 1% to 66%. Traditionally, AKI assessment relied on classifications such as RIFLE (Risk, Injury, Failure, Loss, and End-stage renal disease) [3], AKIN (Acute Kidney Injury Network) [4], KDIGO (Kidney Disease: Improving Global Outcomes) criteria [5], and CTCAE (Common Terminology Criteria for Adverse Events) [6]. Notably, the KDIGO classification stands as the most frequently employed tool in clinical practice. AKI is a prevalent complication linked to heightened mortality rates in liver diseases, acute myocardial infarction (AMI), and cancer patients [7]. Its occurrence fluctuates based on AKI criteria, disease types, treatments, and patient-specific factors. Furthermore, the frequency of hospital-acquired AKI exceeded that of community-acquired cases [8]. It is noteworthy that AKI frequently manifestes as a severe condition in critically ill cancer patients [9, 10]. Globally, AKI almost affects 10–15% of hospitalized patients, 50% of critically ill patients in the intensive care unit(ICU), 22% of adults, and 34% of children. AKI frequently emerges as a complication during treatments, with its occurrence varying across diverse therapeutic modalities [11]. This discrepancy is exemplified by a range of approaches such as varied surgical techniques [12–18], concurrent chemoradiotherapy [19, 20], allogeneic hematopoietic stem cell transplantation (HSCT) [21], immune checkpoint inhibitors (ICIs) [22, 23], a combination of androgen deprivation therapy (ADT) with definitive radiotherapy [24], and surgery [25]. Of note, 20–50% of patients in cardiac surgery suffer from cardiac surgery-associated AKI, which was associated with postoperative morbidity and mortality [26]. Cardiopulmonary bypass (CPB) also leads to alterations in renal physiology [27]. Contrast-induced AKI (CI-AKI) is a common complication following coronary interventions, which are associated with elevated risks of mortality and morbidity [28]. 11–40% of patients with iodine contrast medium for diagnosis or treatment will develop AKI.

Extensive research has elucidated the intricate and dynamic interplay between cancer and AKI [29–31] (Table 1). Past research has indicated a higher prevalence of AKI in hematologic malignancies when contrasted with solid tumors. The predominant cancer-associated AKIs encompassed the hematological system (myeloma, leukemia, lymphoma), urinary system (urinary tract cancer, bladder cancer), and digestive system (hepatocellular carcinoma (HCC), gastrointestinal cancer) [8, 32–34]. The existence of nephrotoxic effects in recipients of cancer therapeutics continues to serve as a significant limitation to the utilization and effectiveness of cancer treatments. AKI emerges as a crucial prognostic indicator for adverse outcomes in cancer patients [33]. Notably, in HCC individuals receiving transcatheter arterial chemoembolization (TACE), the presence of AKI amplifies the risk of mortality by 4.74 times compared to those unaffected by AKI [17]. Moreover, post-transplant allogeneic HSCT-induced AKI significantly escalates 5-year non-relapse mortality (*P* < 0.001) [21]. Studies reveal that AKI in cancer patients is associated with a sevenfold increase in mortality [35]. Particularly in lung cancer cases, AKI predicts a 5-year survival rate of only 46.9% [36]. Consequently, AKI stands as a pivotal limitation in clinical therapy, emphasizing the urgent need to elucidate the underlying mechanisms of AKI for its early detection, prevention, or mitigation.

**Table 1:** The prevalence and contributing factors of AKI among cancer patients

**Country **	**Published year**	**Cohort**	**Therapy**	**AKI criteria**	**Incidence of AKI**	**Risk factors**	**References**
China	2019	136,756 adult cancer patients	-	KDIGO	7.5%	age, increased baseline serum creatinine, shock and urinary tract obstruction	[8]
China	2023	4107 patients were encompassed within the 8 studies focusing on critically ill patients with malignant tumor	-	KDIGO	52.0%	sepsis and hypovolemia	[9]
China	2023	292874 patients with malignant tumors	-	KDIGO	24.0%	sepsis and hypovolemia	[9]
United Kingdom	2020	429 patients with a hematological malignancy or solid tumor	-	KDIGO	60.0%	-	[10]
China	2017	2094 esophageal cancer cases	surgery	KDIGO	2.4%	Preoperative serum creatinine level, surgical duration, smoking history and presence of hypertension	[11]
Ireland	2022	1135 esophageal cancer cases	Surgery: 85% involved an open thoracotomy	AKIN	18.3%	Preoperation: age, male sex, BMI, dyslipidemia Post-operation: atrial fibrillation and pneumonia	[12]
Korea	2021	987 colorectal cancer cases	Laparoscopic surgery	KDIGO	8.7%	albumin, diabetes mellitus, BMI, and hypertension	[13]
Korea	2021	2650 colorectal cancer cases	Open surgery	KDIGO	7.7%	albumin, diabetes mellitus, BMI, and hypertension	[13]
Korea	2016	866 bladder cancer cases	radical cystectomy	AKIN	31.1%	-	[14]
Germany	2016	253046 renal cell carcinoma cases	14,303 receiving radical and 3,525 in partial nephrectomy		5.5%	male sex, radical nephrectomy, older age, black race, more contemporary years (2004-2010), higher comorbidities, higher preoperative CKD stage, medicare insurance status	[15]
China	2023	282 patients with advanced ovarian cancer	cytoreductive surgery with hyperthermic intraperitoneal chemotherapy	KDIGO	11.7%	baseline eGFR<60 mL/min/1.73 m^2^, higher cisplatin dose≥70 mg/m^2^, and concurrent medications of ACEI or ARB	[16]
China	2022	3581 HCC cases	TACE	RIFLE AKIN KIDGO	11.9	Age, DM, and the number of TACE	[17]
China	2022	8284 HCC cases	hepatectomy	RIFLE AKIN KIDGO	12%	male gender, age, DM, major resection of the liver, and operation-related transfusion	[17]
Itay	2022	576 UTUC patients	radical nephroureterectomy	A threshold of 60 ml/min for eGFR was used to determine renal decline function at the 6- and 12-month follow-up periods	41.5%	-	[18]
Itay	2023	109 LA-HNSCC patients	concurrent chemoradiotherapy with at least a cumulative dosage of 200 mg/m2 of cisplatin	KDIGO	12.8%	hypertension, baseline eGFR, and therapy with RAASi	[19]
Thailand	2021	509 LA-HNSCC patients	definitive or postoperative cisplatin-based chemoradiotherapy (CRT)	KDIGO	13.4%	cumulative cisplatin dose, delay, dose reduction, termination, and hospitalization	[20]
Japan	2020	114 cases	allogeneic hematopoietic stem cell transplantation	KDIGO	64.9%	Ages ≥ 46 years at the time of transplant and use of three or more nephrotoxic drugs	[21]
China	2023	24048 cases	ICIs	A rise in serum creatinine to at least 1.5 times the baseline value, either confirmed or presumed to have taken place within the preceding 7 days; or an increase in serum creatinine by ≥26.5 µmol/L within 48 hours; or oliguria (urine volume <0.5 ml/kg per hour for 6 hours	5.7%	older age, preexisting CKD, ipilimumab, combined use of ICIs, extra renal irAEs, and PPI, NSAID, fluindione, diuretics and ACEI/ARB	[22]
China	2023	5267 cases	ICIs	KDIGO		male, hypertension, pre-existent use of a diuretic, and PPI usage, extrarenal irAEs, treatment with CTLA-4	[23]
USA	2023	18754 prostate cancer cases	RT + ADT	KIDGO	10.5%	ADT usage	[24]
USA	2023	9114 prostate cancer cases	RT	KIDGO	7.9%	-	[24]
Korea	2018	2363 brain tumor cases	surgery	KDIGO	1.8%		[25]
Canada	2019	163071 primary cancer cases	systemic therapy	-	27 per 1000 person-years	advanced stage, CKD, diabetes, concomitant receipt of diuretics or ACEI/ARBs	[32]
Korea	2019	67,986 cancer patients		KDIGO	33.8%	-	[33]
State of Palestine	2022	638 solid tumors or multiple myeloma cases	-	RIFLE	6.9%	congestive heart failure, chronic kidney disease, sepsis, ICU admission	[35]
Kera	2021	3202 lung cancer cases	-	KDIGO	55.7%	-	[36]

*AKI* Acute kidney injury, *RIFLE* Risk, Injury, Failure, Loss, and End-stage renal disease, *AKIN *Acute Kidney Injury Network, *KDIGO* Kidney Disease: Improving Global Outcomes, *CTCAE* Common Terminology Criteria for Adverse Events, *CKD* Chronic kidney disease, *ACEI* Angiotensin-converting enzyme inhibitors, *ARB* Angiotensin receptor blockers, *PPI* Proton pump inhibitor, *RAASi* Renin–angiotensin–aldosterone system inhibitor, *NSAID* Nonsteroidal anti-inflammatory drug, *DM* Diabetes mellitus, *BMI* Body mass index, *eGFR* Estimating Glomerular Filtration Rate, *irAEs* Immune-related adverse events, *CTLA-4* Cytotoxic T Lymphocyte antigen 4, *ICIs* Immune checkpoint inhibitors, *ICU* Intensive care unit, *RT* radiotherapy, *ADT* Androgen deprivation therapy, *HCC* hepatocellular carcinoma, *LA-HNSCC* Locally advanced head and neck squamous cell carcinoma, *UTUC* Upper tract urothelial carcinoma, *TACE* transarterial chemoembolization

Previous studies have elucidated the intricate molecular interplay between clinical disorders and AKI. Drug-induced AKI is a well-recognized form of nephrotoxicity, recent advances in understanding novel mechanisms such as ferroptosis, mitochondrial regulation, and cell senescence have created new opportunities for addressing AKI. Furthermore, onco-nephrology is a new and evolving subspecialized area which attracted more and more attention. The incorporation of innovative therapeutic modalities like ICIs introduces increased complexity and diversity in managing AKI. Targeting mitochondria or signaling pathways is an emerging approach for AKI. More importantly, AKI not only has adverse effects on organs other than kidneys but also leads to elevated health-care costs. Therefore, subphenotyping is essential to facilitate personalized management strategies.

This review comprehensively overviews the pathophysiology of AKI and further elucidates the pathogenesis of AKI attributed to both clinical conditions and its treatments. Then it expounds on risk factors and causes, which makes a significant contribution by identifying predisposing factors from patient-specific sources. This review focuses on the diagnosis of AKI, finally summarizes therapeutic interventions, evaluates therapeutic gaps, and highlights the early diagnosis of AKI and how timely intervention paves the way to prevent and alleviate AKI.

## Risk factors and causes

### Common risk factors for acute kidney injury

The primary risk factors for AKI encompass volume depletion, haemodynamic instability, inflammation, exposure to nephrotoxic agents, and mitochondrial dysfunction. In cases of obstructive nephropathy, the density of lymphatic vessels is notably increased, acting as an independent predictor of renal function decline [37]. In addition, underlying co-morbidities such as type 2 diabetes mellitus (T2DM) and hypertension can further increase the risk of AKI. It has been reported that rhabdomyolysis, surgical procedures, and mechanical ventilation are all related to an increased risk of AKI [38]. As representative diseases, cancer is closely linked with the enhancement of AKI, Table 1 and Supplementary Table 1 showed the cancer patients-related risk profile for susceptibility to AKI.

Growing evidence indicates that patient variables significantly increase the risk of AKI in individuals with cancer or undergoing cancer-related therapies [32] (Table 1 and Supplementary Table 1). Advanced age and male gender are commonly recognized as independent risk factors for AKI in cancer patients. Additionally, conditions such as Diabetes mellitus (DM), chronic renal disease, or CKD are frequently reported as prevalent comorbidities. Factors such as metabolic syndrome, nutritional status, hypertension, dyslipidemia, increased body mass index (BMI), and lower albumin levels are considered key contributors to the heightened risk of AKI development [12, 13, 25]. A variety of organ-related conditions, such as shock, atrial fibrillation, and pneumonia, which were present prior to treatment, have been identified as independent predictors of AKI onset [8, 11, 12]. Notably, congestive heart failure, sepsis, and hypercalcemia are associated with substantially increased odds of AKI development in patients with solid tumors or multiple myeloma [35]. Baseline serum creatinine levels and estimated glomerular filtration rate (eGFR) also serve as valuable independent risk indicators for AKI occurrence in esophageal cancer patients. Furthermore, tobacco consumption is linked to a 3.029-fold increased risk of AKI in individuals with esophageal cancer [11].

Several researchers have delved into the influencing factors of AKI in cancer patients undergoing surgery, radiotherapy, immunotherapy, and HSCT. The quality and efficacy of treatment can directly impact outcomes and the risk of AKI, such as the duration of operation for esophageal cancer [11], the quality of TACE or the volume of hepatectomy-related transfusions contributing to AKI in HCC [17], and radical nephrectomy for renal cell carcinoma (RCC) [15]. Concurrent medications have emerged as significant predictors of AKI in cancer patients, with noteworthy associations observed between AKI and the simultaneous use of diuretics, angiotensin-converting enzyme inhibitors (ACEI), angiotensin receptor blockers (ARB) [16], and Renin–angiotensin–aldosterone system inhibitor (RAASi) treatment [19]. Notably, the administration of three or more nephrotoxic drugs was linked to an increased risk of AKI in allogeneic HSCT patients [21]. In individuals receiving ICIs, the use of proton pump inhibitors (PPIs), nonsteroidal anti-inflammatory drugs (NSAIDs), fluindione, diuretics, and ACEI/ARBs was associated with a heightened risk of AKI [22, 23]. Immune-related adverse events(irAEs) are common side effects of ICIs, with extrarenal irAEs linked to ICIs-induced AKI. In addition to the aforementioned factors, the occurrence of AKI was found to be dose-dependent, particularly in cases of higher cisplatin doses ≥ 70 mg/m^2^. Collectively, certain clinicopathological features, concurrent medication use, and underlying comorbidities are recognized as predisposing factors for AKI in cancer patients, aiding in the assessment of AKI occurrence.

### Types and causes of acute kidney injury

Traditionally, AKI can be categorized into three types based on its pathophysiological mechanisms and the sites of anatomical injury: pre-renal, intrinsic renal, and post-renal injury. Specifically, intrinsic renal injury is implicated in renal AKI, which may involve the renal tubules, interstitium, renal vessels, and glomeruli. In general, AKI present at admission or developing within 48 h of admission is generally classified as community-acquired AKI, whereas AKI developing after this period is classified as hospital-acquired AKI. Broadly speaking, aetiological subtypes of AKI included ischemia-induced AKI, infection-related AKI, sepsis-induced AKI, contrast-induced AKI, and drug-induced AKI. Sepsis-induced AKI can be categorized into two distinct sub-phenotypes: AKI subphenotype 1(AKI-SP1) is characterized by a reparative, regenerative phenotype, and AKI subphenotype 2(AKI-SP2) is marked by an immune and inflammatory phenotype associated with blood bacteremia [39]. Therefore, the type of AKI varies in different clinical standards or etiology.

#### The causes of pre-renal acute kidney injury

Renal ischemia is a central mechanism underlying pre-renal AKI. Various factors can contribute to pre-renal AKI, including gastrointestinal symptoms, bleeding and tumor thrombus, hepatorenal syndrome, paraneoplastic syndrome, hypercalcemia, and nephrectomy-induced ischemic injury mediated by cancer. Additionally, treatment-related factors such as gastrointestinal effects and complications, including veno-occlusive disease (VOD) or sinusoidal obstruction syndrome following HSCT, can all lead to pre-renal AKI associated with renal hypoperfusion. Pre-operative dehydration may also be associated with the development of postoperative AKI [40].

#### The causes of intrinsic acute kidney injury

Intrinsic renal injury is the predominant cause of AKI. Intrinsic AKI, also referred to as intrarenal AKI, originates from damage within the kidney itself and can impact various renal structures, including the glomeruli, which may lead to glomerulonephritis; the tubules, which can result in acute tubular necrosis (ATN); and the interstitium, which can cause acute interstitial nephritis (AIN).

In patients with multiple myeloma (MM) [41] and lupus nephritis [42], free light chain casts can obstruct renal tubules, contributing to AKI. Other causes of intrinsic renal damage include conventional platinum-based chemotherapy and novel targeted therapy, or immunotherapy agents used in cancer treatment. The majority of intrinsic AKI cases are induced by nephrotoxic substances such as aminoglycosides, vancomycin, and amphotericin B. Additionally, renal ischemia, which may result from shock, surgical complications, or hemorrhage, primarily leads to tubular injury. Interstitial damage can also be caused by nephrotoxic drugs like beta-lactam antibiotics and NSAIDs, infections, and other autoimmune conditions such as systemic lupus erythematosus (SLE). In addition, vascular-related factors such as thrombotic microangiopathy (TMA) also induced AKI. Renal damage is commonly associated with TMA. Following an allogeneic hematopoietic cell transplant (HCT), endothelial damage serves as the primary instigator of AKI. This damage sets off a chain reaction that activates the coagulation system, resulting in thrombin production, fibrin formation, and platelet clumping. The development of AKI is also correlated with the activation of the coagulation system, as evidenced by elevated plasma levels of Plasminogen activator inhibitor-1 (PAI-1), tissue plasminogen activator (tPA), and D-dimer [43]. The rapid decline in renal function seems to be associated with factors like a neoplastic thrombus that causes blockage in the renal vein [44], clotting disorders [45], and thrombocytopenia. TMA is an uncommon complication of gemcitabine treatment [46]. Furthermore, proteasome inhibitors have the potential to reduce nuclear factor kappa-light-chain-enhancer of activated B cells (NF-κB) levels in the nucleus, leading to VEGF inhibition, which is identified as one of the potential pathological factors contributing to TMA associated with AKI, potentially predisposing to TMA [47]. Hypoxic AKI arises from a mismatch in renal oxygenation, characterized by reduced oxygen supply and heightened oxygen consumption for tubular transport [194].

#### The causes of post-renal acute kidney injury

Urinary tract obstruction (UTO) is a primary cause of post-renal AKI, with anuria or oliguria often occurring rapidly following the obstruction. Tumor-induced obstruction or invasion of the urinary system could lead to local or post-renal obstruction and compromised renal function.

### Special types of acute kidney injury

Aside from the typical risks and causes, various unique conditions are present in diverse clinical settings, such as AKI associated with coronavirus disease 2019 (COVID-19), pregnancy-related AKI (pAKI), neonatal AKI, tumor lysis syndrome (TLS) induced AKI, and sepsis-induced AKI.

#### COVID-19-associated acute kidney injury

COVID-19 is associated with a heightened risk of AKI [48]. A retrospective longitudinal multicenter cohort study indicated that patients with COVID-19-related AKI were generally younger, had a higher baseline eGFR, worse baseline comorbidity scores, elevated markers of illness severity, and longer hospital stay compared to those suffering from influenza-related AKI or other types of AKI [49]. In another cohort study involving veterans of African ancestry hospitalized with COVID-19 infection, it was found that variants of apolipoprotein L1 (APOL1) were linked to increased likelihood of AKI, greater AKI severity, and higher mortality rates, even among individuals with prior normal kidney function [50]. Substantial alterations in circulating levels of immune mediators [51] occurred in COVID-19-associated AKI, suggesting exacerbation of the immune response appears to play a role in the development of AKI. Given that long COVID has the potential to impact nearly every organ system [52], hence the relationship between AKI and long COVID has piqued our interest.

#### Pregnancy-related acute kidney injury

AKI during pregnancy can emerge from diverse causes and at different gestational stages. Prerenal and intrinsic AKI resulting from hemorrhage, which induces hypovolemia and renal ischemia, are the most prevalent types [53]. In the first and second trimesters, prerenal AKI is more frequent, often caused by septic abortions, hemorrhage, and hyperemesis gravidarum. Both hyperemesis gravidarum and septic abortions can result in hypovolemia and metabolic alkalosis, subsequently causing hypotension and renal ischemia, and ultimately resulting in AKI. In the third trimester, intrarenal AKI is the most frequent type of AKI in pregnancy, accounting for approximately 75% of cases. The primary causes of intrarenal AKI during this period are hypertensive disorders of pregnancy, such as preeclampsia and HELLP syndrome, as well as other rarer etiologies like TMA and acute fatty liver of pregnancy. Although rare, postrenal AKI may also occur during delivery or parturition.

#### Neonatal acute kidney injury

Neonatal AKI is a prevalent and independent risk predictor of increased mortality and prolonged hospitalization. Neonates, like pediatric and adult patients, can also be profoundly impacted by AKI. A meta-analysis compassing 201 studies from 45 countries systematically assesses the worldwide incidence of AKI in neonates and uncovered the incidence of any stage AKI was 30% and AKI-associated mortality was 30% [54]. Neonatal recurrent AKI was independently associated with longer LOS when compared with a single AKI episode [55].

#### Tumor lysis syndrome induced acute kidney injury

**Fig. 1:**
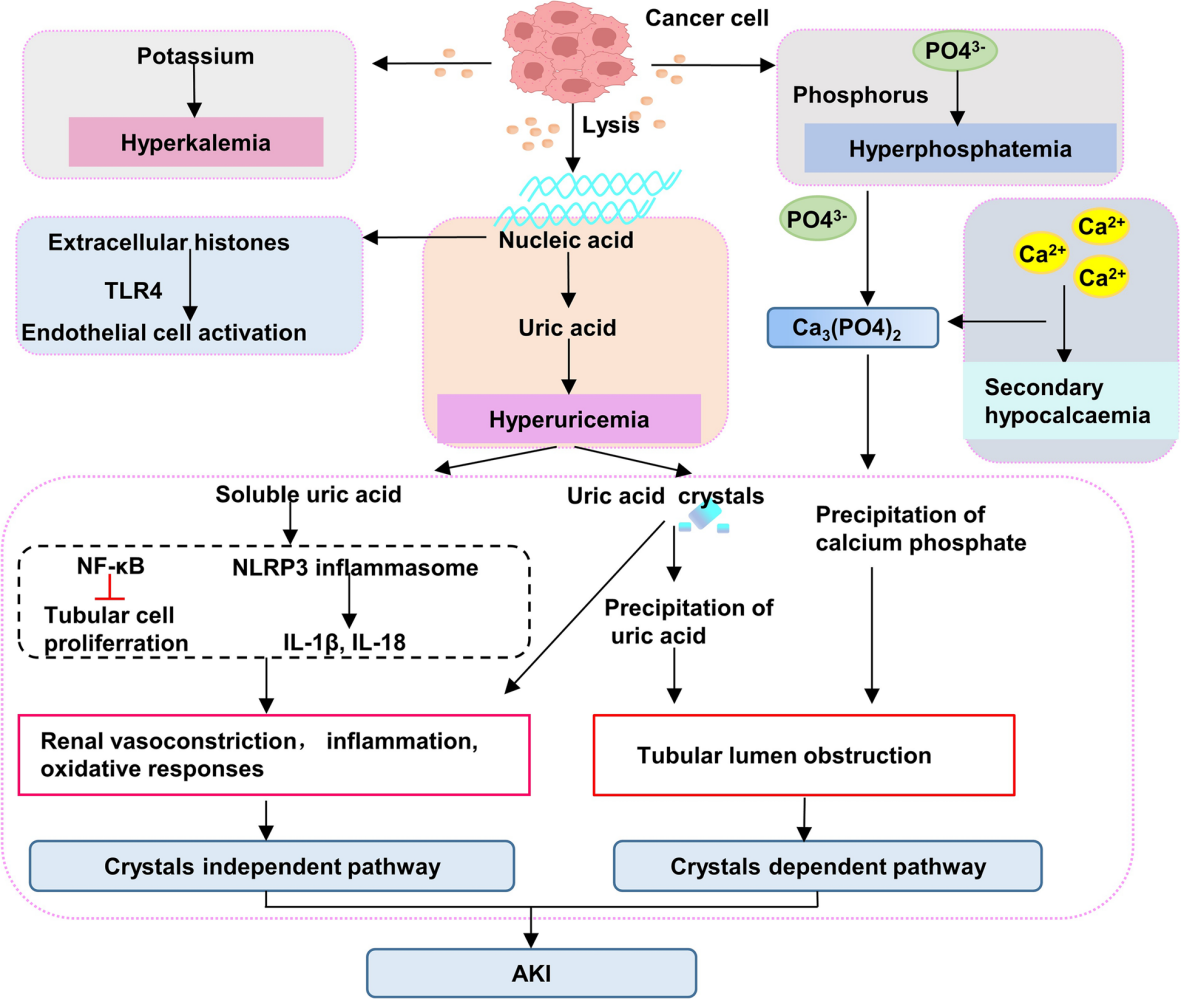
The mechanism of tumor lysis syndrome (TLS). TLS led to electrolyte disturbance, such as hyperphosphatemia, hyperkalemia, and hyperuricemia. Further, hyperuricemia caused AKI by crystal-dependent injury or crystal-independent injury. Note: AKI: Acute kidney injury; NF-κB:Nuclear factor kappa-light-chain-enhancer of activated B cells. (figure was created in BioRender. Xu, X. (2025) https://BioRender.com/f8wgo85)

TLS is a dynamic metabolic complication associated with a heightened mortality rate, particularly evident in patients undergoing chimeric antigen receptor-T (CAR-T) cell therapy and bispecific T cell-engaging antibodies. TLS-induced AKI affects up to 64% of TLS patients, with an increased likelihood in cases of proliferative and/or chemosensitive malignancies harboring sizable tumor burdens. The abrupt and extensive breakdown of tumor cells leads to the release of intracellular components like potassium, phosphorus, and nucleic acids into the bloodstream, triggering TLS **(**Fig. 1**)**. The excessive glomerular filtration capacity in normal kidneys can lead to blood clotting and metabolic disturbances like hyperphosphatemia, hyperkalemia, and hyperuricemia. TLS-induced AKI is characterized by crystal-driven damage caused by the deposition of uric acid and calcium phosphate in renal tubules [56, 57], triggering inflammatory and pro-oxidative responses that harm the tubular epithelium. Elevated serum levels of uric acid are associated with hemodynamic changes resulting from poor autoregulation and renal vasoconstriction [58]. Conversely, uric acid may also contribute to AKI through mechanisms independent of crystal formation [59]. Besides, the increased release of extracellular histones during TLS led to significant endothelial changes in the mouse model. The activation of endothelial cells through Toll-like receptor 4 (TLR4) is involved in the pathways of histone-mediated damage. Heparin mitigates endothelial dysfunction during TLS by inhibiting extracellular histones [59]. It is crucial to differentiate between TLS and spontaneous TLS (STLS). STLS is a rare oncologic emergency, particularly in tumors with a larger burden and higher proliferation rates [60, 61], triggered by extensive cancer cell lysis or necrosis without a specific cause [62], leading to normal or slightly elevated phosphate levels which are attributed to the utilization of phosphate in rapid tumor cell proliferation.

#### Sepsis-induced acute kidney injury

Infections are the primary cause of non-relapsed mortality after treatment with CAR-T cell therapy and bispecific T-cell-engaging antibodies. A multitude of factors, encompassing local inflammation, metabolic reprogramming [63], and microvascular endothelial dysfunction [64], contribute to the development of sepsis-induced AKI. Yang Y et al. suggest a significant link between septic AKI and certain cancer types [64], including bladder and retroperitoneal tumors. Surgical therapy increases the risk of inadequate organ perfusion during procedures. After undergoing nephrectomy, patients may encounter complications like intra-abdominal hypertension or abdominal infections. Moreover, AKI in tumor-related thrombotic microvascular disease and septic coagulation dysfunction can occur due to renal microvascular thrombosis with endothelial edema and microvascular blockage. The presence of these factors significantly heightens the likelihood of septic AKI in cancer patients. Taken together, grasping the risks and causes of AKI lays the foundation for a more precise diagnosis of the condition.

## Pathophysiology of acute kidney injury

The causes of AKI are highly complex and varied [65]. Factors such as infection [66], sepsis [67, 68], hypoxia [69], nephrotoxic drugs [70], rhabdomyolysis [71] and numerous others can all lead to AKI. While AKI is frequently multifactorial, haemodynamic instability, sepsis, and drug toxicity are frequently implicated. Regardless of the specific cause, multiple pathophysiological processes unfold simultaneously and in sequence, including endothelial dysfunction, alteration of the microcirculation, tubular injury, obstruction, venous congestion, and intrarenal inflammation. Epidemiologically, community-acquired AKI is more commonly of pre-renal origin and predominantly affects older individuals or those with pre-existing health conditions. It is often triggered by dehydration or the use of medications that compromise glomerular blood flow. Conversely, AKI that is acquired in the hospital setting is more often intrinsic in nature and typically presents with greater severity. AKI frequently occurs as a secondary issue among hospitalized patients, rather than being the main reason for their admission. It is often triggered by damage or impaired function in organs outside the urinary system. Sepsis stands out as a significant cause of AKI acquired during hospitalization, contributing to approximately 30–50% of such cases. In the context of sepsis, microbial constituents and substances released by the host function as alert signals, or alarmins, that engage with pattern recognition receptors (PRR). The renal endothelium, tubular epithelial cells (TECs), and immune cells all express PRR capable of recognizing a broad spectrum of injury-related molecular patterns. Stimulation of PRR elicits pro-inflammatory changes in renal cells and sets off pathways leading to programmed cell death. Meanwhile, immune cells are recruited to the location where alarmins are released, thereby fueling local inflammatory responses. IRI is likely the most thoroughly investigated experimental model of AKI. In clinical settings, it typically arises from severe renal hypoperfusion due to blood loss, septic shock, or other anatomical abnormalities that compromise renal blood flow. Multiple interacting etiologies contribute to AKI in clinical settings such as liver disease, heart failure, cancer, etc. Therapeutic manners also cause nephrotoxic injury including AKI. The pathophysiology of AKI secondary to cardiac surgery (coronary artery bypass grafting) might include hemodynamic instability, renal ischemia–reperfusion, systemic inflammatory response, or exposure to nephrotoxic medications. Exposure to the CPB circuit also leads to complement activation, hemolysis, and systemic inflammation. In this section, we mainly focus on clinical disorders, therapy-related nephrotoxicity, inflammation, and oxidative stress to share the underlying pathophysiological mechanisms of AKI.

### The pathophysiological mechanism of acute kidney Injury induced by clinical conditions

#### The pathogenesis of acute kidney injury induced by liver disease

Individuals with cirrhosis are at an increased risk of developing all forms of AKI, including hepatorenal syndrome (HRS)-related AKI and non-HRS-AKI. HRS-AKI, a distinct form of AKI in individuals with advanced cirrhosis and ascites, is associated with an exceptionally high mortality risk. It stems from reduced renal blood flow due to changes in the arterial circulation and the overactivity of the body’s natural vasoactive systems including the renin–angiotensin–aldosterone system(RAAS), the sympathetic nervous system, and arginine vasopressin [72]. Systemic inflammation significantly contributes to the development of neurohumoral and vasodilatory imbalances, which subsequently result in functional AKI (HRS-AKI). In Japanese patients with cirrhosis, an imbalance in amino acids is strongly correlated with the development of AKI [73]. In the case of non-HRS-AKI, the predominant causes of AKI in patients with cirrhosis include hypovolemia, spontaneous bacterial peritonitis (SBP), bacterial infections (other than SBP), sepsis, upper gastrointestinal bleeding, and shock. Infections and sepsis can reduce blood flow to the kidneys. This can cause kidney damage in cirrhosis patients, who are already sensitive to changes in fluid volume. In the setting of cirrhosis or chronic liver disease, inflammation may result from damage-associated molecular patterns (DAMPs) in hepatocytes and the weakening of gut immunity due to pathogen-associated molecular patterns (PAMPs).

#### The pathogenesis of AKI induced by cardiovascular disease

Cardiorenal syndrome (CRS) is a complex, bidirectional condition marked by interdependent dysfunction of the heart and kidneys whereby the dysfunction of one organ, whether acute or chronic, can lead to dysfunction of the other organ in an acute or chronic manner. The underlying mechanism may involve hemodynamic dysregulation, a reduction in effective circulating volume, renal venous congestion, activation of the sympathetic nervous system and renin–angiotensin–aldosterone system (RAAS), inflammation, neurohormonal activation, and an imbalance in reactive oxygen species(ROS)/nitric oxide synthase(NOS) [74]. AKI in patients with atrial fibrillation might be induced by ATN and embolic events caused by hemodynamic instability and anticoagulation-related nephropathy.

#### Kidney involvement induced by cancer

Tumor infiltration of the kidney or kidney involvement can lead to AKI by causing compression and obstruction of the urinary tract, such as in cases of bladder cancer [14] and hematological malignancies [75]. 90% of lymphoma patients demonstrate bilateral interstitial lymphoma cell infiltration and an increase in renal size on radiographic imaging [76]. Peripheral T-cell lymphoma, not otherwise specified (PTCL-NOS), infrequently infiltrates the kidneys but can also cause AKI due to lymphoma cell invasion of the renal interstitial space [77]. Furthermore, AKI can occur from tubular compression and disruption of renal microcirculation because of tumor infiltration. Lymphomatous invasion of the kidneys (LIK) may be presented with hematuria and proteinuria. Invasive cancers disrupting the renal parenchyma led to AKI by affecting the glomerular, tubulointerstitial, and microvascular structure. In patients with MM, light chain cast nephropathy (LCCN) represents a pivotal myeloma-related event contributing to AKI [41]. The presence of filtered free light chains (FLCs) within the glomerulus leads to excessive light chain excretion into the urine, which in turn compromises the resorptive capacity of the proximal tubules. Subsequently, the excess monoclonal FLCs interact with the Tamm Horsfall protein in the loop of Henle, leading to the formation of light chain casts. The obstruction of the tubules by these casts can result in tubular rupture and injury, which is often initiated by an immune response. Additionally, the generation of hydrogen peroxide by the FLCs further intensifies the renal injury, stimulating the nuclear factor kappa-light-chain-enhancer of activated B cells (NF-κB) and apoptosis signal-regulating kinase 1 (ASK1), as well as the Janus kinases(JAK) and signal transducer and activator of transcription proteins (STATs) pathway [78]. These processes drive tubulointerstitial fibrosis, inflammation, and apoptosis, amplifying the damage beyond mere tubular obstruction [79, 80]. Both the physical blockade of the distal tubules and the FLC-induced injury to the proximal tubules collectively contribute to the development of AKI.

AKI resulted from cancer metastasis to the kidney, involving non-small cell lung cancer (NSCLC), small cell lung cancer (SCLC), and HCC [81, 82]. Renal carcinomatous lymphangitis also played a role in AKI occurrence [83]. Lymphostasis arises from compromised lymph circulation, impacting the removal of interstitial fluid. Failure in lymphatic vessels’ fluid clearance from the interstitial space hinders its return to the bloodstream. The release of cytokines and growth factors by damaged tubular cells triggers interstitial inflammation, accumulation of extracellular matrix, and eventual fibrosis. Disrupted calcium metabolism in cancer patients, including those with squamous cell carcinoma, renal carcinoma, genitourinary malignancies, breast cancers, pancreatic cancer [84], metastatic pancreatic neuroendocrine tumor [85], and human T-cell leukemia virus type 1-associated adult T-cell leukemia/lymphomas, often leads to AKI. It is important to note that elevated levels of calcium in the blood can lead to marked volume depletion through the stimulation of the calcium sensor located in the thick ascending limb of the loop of Henle, resulting in physiological effects resembling those induced by furosemide. Hypercalcemia triggers vasoconstriction of the afferent arteriole, leading to a decrease in intra-glomerular pressure. Furthermore, it results in the precipitation of calcium phosphate crystals and tubule blockage. Malignancy-associated hypercalcemia has been identified to be driven by three primary mechanisms [86, 87]. Firstly, the abnormal secretion of parathyroid hormone-related peptide (PTHrP), particularly notable in squamous cancers, plays a crucial role in hypercalcemia by binding to the type I PTH/PTHrP receptor (PTHR-1) and activating pathways that promote increased calcium resorption from bones and reabsorption in the kidneys [88]. PTHrP stimulates bone resorption by activating osteoblasts to produce receptor activators of nuclear factor-kappa ligand (RANKL) and osteoclast precursors. Secondly, hypercalcemia can result from local osteolysis induced by tumor invasion of bone. Thirdly, absorptive hypercalcemia stems from the overproduction of vitamin D by malignancies in a PTH-independent manner, facilitated by the activation of 25-hydroxyvitamin-D3 1α-hydroxylase by tumor-associated macrophages. Moreover, hypercalcemia serves as a significant prognostic indicator for malignancy [89]. It is crucial to distinguish pseudo-hypercalcemia stemming from abnormal immunoglobulins [90].

Additionally, as renal complications in many diseases, including COVID-19, inflammatory bowel disease, lupus nephritis in childhood-onset SLE, age-related diseases such as Alzheimer's and Parkinson's disease, AKI remains the similar multiple interaction of pathophysiological mechanism following some other diseases.

### The pathophysiological mechanism of acute kidney injury induced by therapeutic nephrotoxicity

Treatment manners, including surgery and medications (Supplementary Table 2), are leading causes of AKI in hospitalized patients, especially in critically ill settings. The mechanisms underlying drug-associated AKI are diverse, including direct tubular damage, tubulointerstitial inflammation, and intratubular crystal precipitation. Drug-induced nephrotoxicity can result in AKI via one of three main mechanisms: Initially, when drugs or their metabolites interact with the apical surface, traverse through it, or are secreted from the basolateral surface into the tubular lumen, they can induce dose-dependent proximal tubular injury and ATN. Second, tubular obstruction caused by crystals or casts containing drugs and their metabolites, is also dose-dependent. Third, interstitial nephritis is triggered by drugs and their metabolites, which is dose-independent. With the emergence of novel therapeutic tools like immunotherapy, it led to nephrotoxicity. Similarly, this section expounds on the recent developments regarding the pathogenesis of nephrotoxicity-induced AKI.

#### Platinum-based chemotherapy and acute kidney injury

##### Excessive accumulation of platinum in proximal tubule epithelial cells

**Fig. 2:**
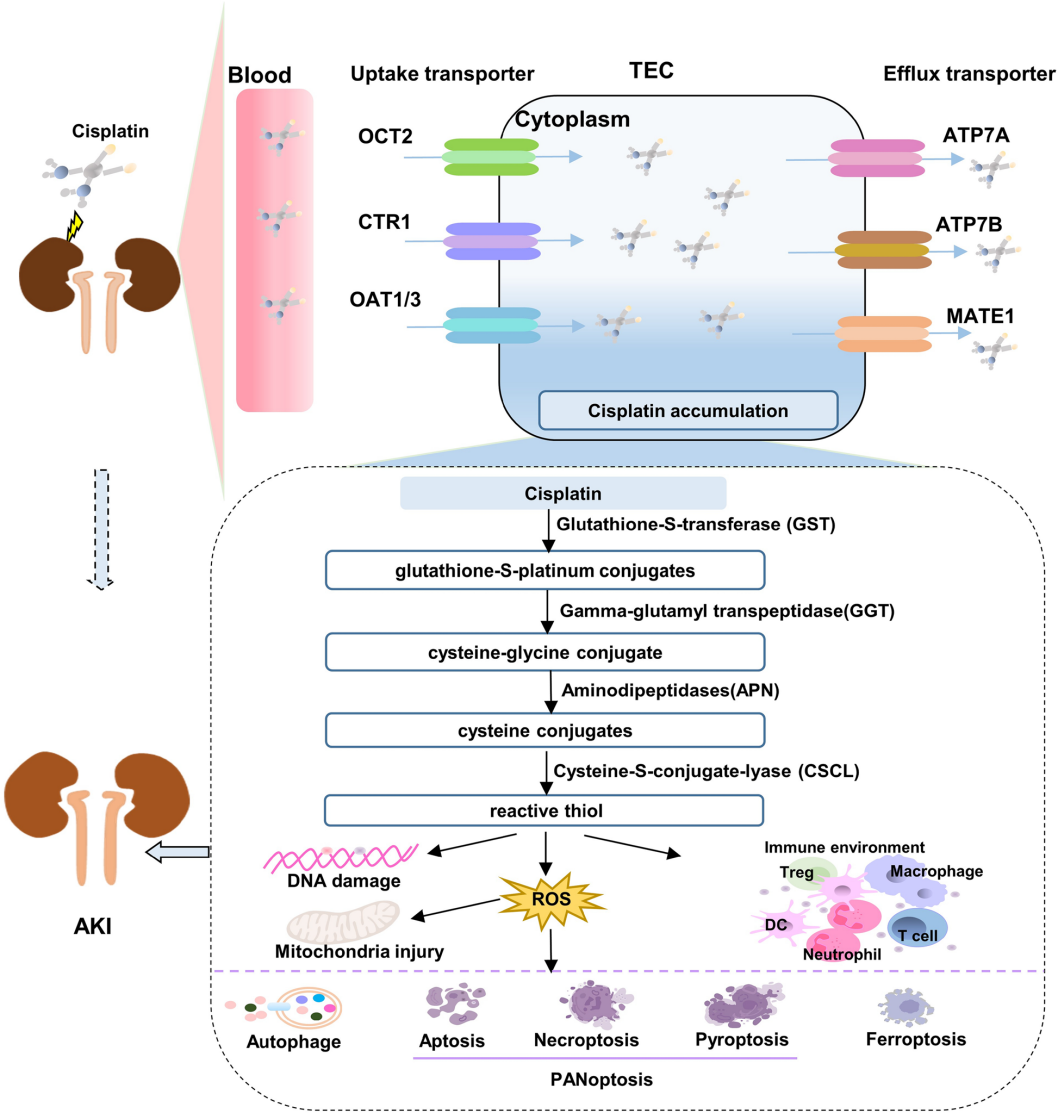
The relationship between cisplatin and AKI. Cisplatin was absorbed and transported into the TEC by various of anion transporters resulting in accumulation of cisplatin. Then accumulated cisplatin metabolites into reactive thiol and induced AKI by diverse mechanisms such as DNA damage, ROS, mitochondria injury, immune environment disorders, and finally cell death. Note: AKI: Acute kidney injury; TEC: Tubule epithelial cells; MATE 1: Multidrug and toxin extrusion protein 1; OCT2: Organic cation transporter 2; MRPs: Multidrug resistance-associated protein; ATP7A: P-type copper transporting ATPases; OAT1/3: Organic anion transporter 1 and 3; CTR1: Copper transporter 1. (figure was created in BioRender. Xu, X. (2025) https://BioRender.com/f8wgo85)

Chemotherapy represents a conventional approach to treating cancer. Since 1978, cisplatin has occupied a pivotal role in chemotherapy regimens for various solid tumors, with cisplatin-induced AKI being an extensively studied mechanism (Fig. 2). The process of cisplatin being taken up by proximal tubule epithelial cells (PTEC) and being converted into a potent nephrotoxin initiates the development of cisplatin-induced nephrotoxicity, including AKI, through a transporter-dependent mechanism at the cellular membrane. Transporters involved in this process, such as organic cation transporter 2 (OCT2), copper transporter 1 (CTR1), and the less investigated volume-regulated anion channels (VRAC), are predominantly localized in the basolateral membrane of PTEC and play a crucial role in facilitating the accumulation and transport of platinum into kidney cells. Among these transporters, OCT2 has been extensively studied as a significant contributor to cisplatin uptake by modulating cellular sensitivity and urinary excretion of cisplatin, leading to 30% of nephrotoxicity [91]. Furthermore, genetic variations or deficiencies in OCT2 can influence its phenotypic activity, predisposition to toxicity, and response to drug treatments. Research has shown that a specific nonsynonymous single-nucleotide polymorphism (SNP), located in exon 4 of the OCT2 gene (rs316019), is associated with reduced cisplatin-induced nephrotoxicity in Caucasian cancer patients [91, 92]. Consequently, OCT2 has been suggested as a potential target for protective therapeutic approaches against cisplatin-induced nephrotoxicity. Organic anion transporters (OATs) facilitate the entry of hydrophilic anions into cells through secondary or active transport processes, thereby maintaining the body's anion equilibrium. Furthermore, the elimination of cisplatin into the urine is governed by apically localized efflux transporters present in both proximal and distal tubules. These transporters comprise multidrug and toxin extrusion protein 1 (MATE1), multidrug resistance-associated proteins (MRPs), and P-type copper transporting ATPases (ATP7A). Tubular injury leads to the accumulation of platinum within the tubules, resulting in reduced glomerular filtration rate (GFR) and delayed urinary excretion of cisplatin [93]. Magnesium plays a crucial role as an essential modulator of cisplatin transporter expression [94]. A deficiency in magnesium following cisplatin injection reduces the expression of efflux transporters, leading to increased accumulation of cisplatin in tubular cells, thereby exacerbating the severity of AKI [95]. Collectively, these findings suggest a link between AKI and the abnormal accumulation of cisplatin in tubular cells.

##### DNA damage

Deoxyribonucleic acid (DNA) damage and the DNA damage response or repair (DDR) are critically implicated in cisplatin-induced AKI. Cisplatin accumulation in PTEC can form cross-links with DNA, disrupting its double-helical structure. Mixed lineage leukemia 1 (MLL1) is identified as a significant histone H3 lysine 4 methyltransferase and acts as a novel regulator of DDR, promoting apoptosis in cisplatin-induced renal proximal tubular cells and AKI. Specifically, MLL1 interacts directly with WD repeat domain 5(WDR5) to initiate DDR, involving phosphorylated ataxia telangiectasia mutated (ATM) kinases/ATM and Rad-3-related (ATR) kinases/and checkpoint kinase(Chk), and the expression of γ-H2AX. Subsequently, it activates p53 to induce cell cycle arrest and modulate E-cadherin expression, leading to apoptosis through activation of caspase-3 in murine renal proximal tubular cells [96]. Promotion of AKI by cisplatin occurs via the upregulation of apurinic/apyrimidinic endonuclease 2 (APE2), resulting in subsequent dysfunction of myosin heavy-chain 9 (MYH9) in the mitochondria of proximal tubule cells due to an additional role of APE2 in DDR [97]. In cisplatin-induced AKI, the reduction in miR-155 levels attenuates TEC apoptosis and DNA damage by upregulating telomeric repeat binding factor 1 (TRF1) and cyclin-dependent kinase 12 (CDK12), ultimately preventing telomere dysfunction and genomic DNA damage [98]. Cisplatin not only impacts mitochondrial DNA but also preferentially binds to proteins in the mitochondrial membrane, particularly the voltage-dependent anion channel [99, 100].

##### Oxidative stress and mitochondrial dysfunction

**Fig. 3:**
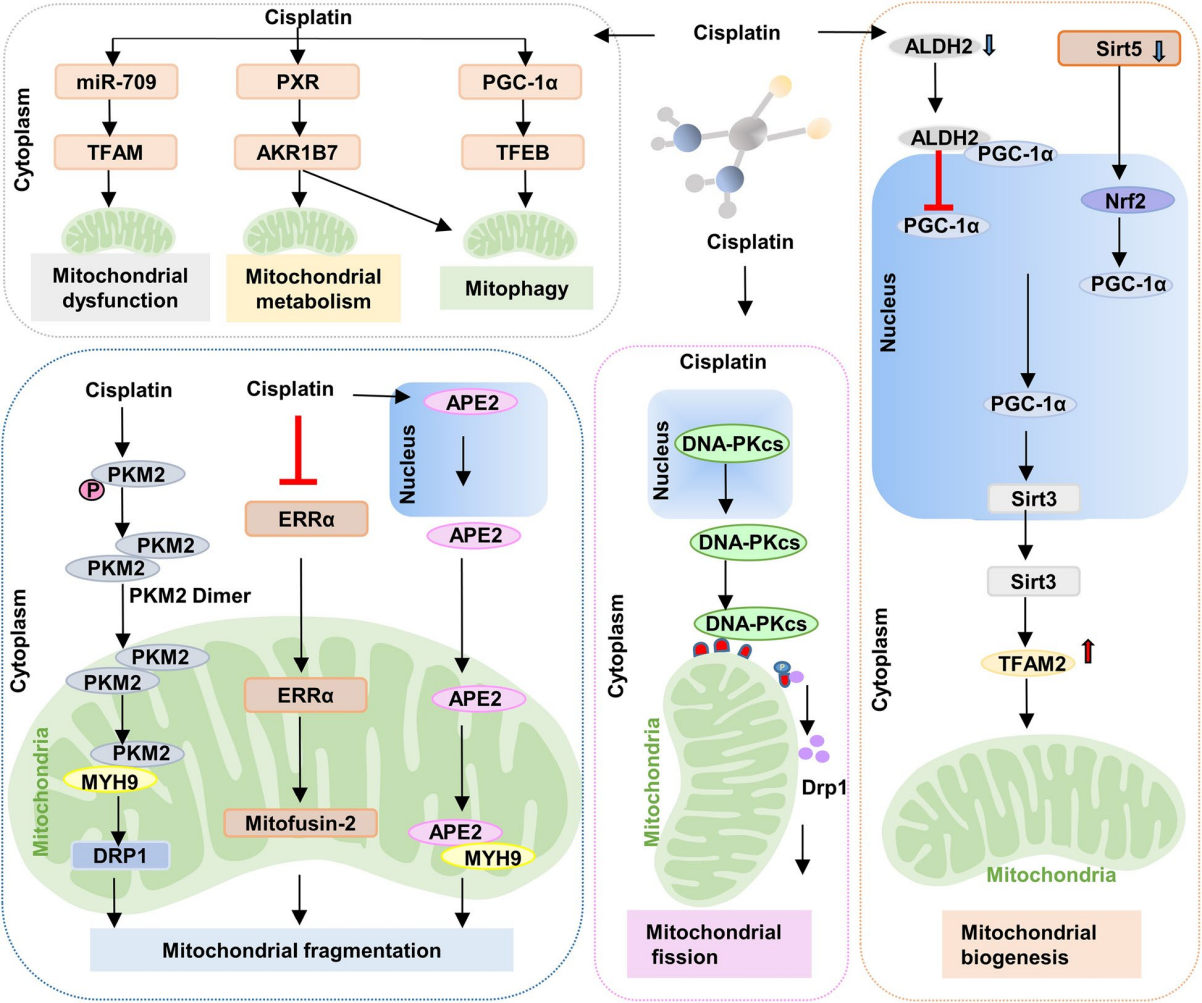
The molecular mechanism of cisplatin-induced AKI by various types of mitochondrial injury. It included mitochondrial dysfunction, fragmentation, fission, biogenesis, mitophagy, regulating mitochondrial metabolism disorder. Note: AKI: Acute kidney injury; ERRα: Estrogen-related receptor alpha; APE2: Apurinic/apyrimidinic endonuclease 2; MYH9: Myosin heavy-Chain 9; TFAM: Mitochondrial transcription factor A; ALDH2: Aldehyde dehydrogenase 2; PGC-1α: Peroxisome proliferator-activated receptor-gamma coactivator 1-alpha; TFEB: Transcription factor EB; DNA-PKcs: DNA-dependent protein kinase catalytic subunit; DRP1: Dynamin-related protein 1; PXR: Pregnane X receptor; AKR1B7: Aldo–keto reductase family 1, member B7; NRF2: Nuclear erythroid 2-related factor 2; Sirt3: Sirtuin 3; Sirt5: Sirtuin 5; PKM2:Pyruvate kinase M2. (figure was created in BioRender. Xu, X. (2025) https://BioRender.com/f8wgo85)

Cisplatin-induced AKI is marked by oxidative stress, which stems from mitochondrial dysfunction and the accumulation of ROS [101]. These ROS interfere with a multitude of signaling pathways, including mitogen-activated protein kinase (MAPK), phosphatidylinositol 3-kinase (PI3K), nuclear factor erythroid 2-related factor 2 (Nrf2), iron metabolism, DDR, and cell death pathways [102]. Notably, the significant role of cisplatin-induced mitochondrial dysfunction as a key contributor is evident [103–108] (Fig. 3). In addition, mutations in transcription factors crucial for mitochondrial biogenesis, such as estrogen-related receptor alpha (ERRα) [109], and mitochondrial transcription factor A (TFAM) [110], have been linked to mitochondrial dysfunction exacerbating cisplatin-induced AKI. Sirtuin3 (Sirt3), the principal deacetylase residing in the mitochondrial matrix, is crucial for modulating mitochondrial function. In a murine model of cisplatin-induced AKI, the absence of Sirt3 led to the dysregulation of fatty acid oxidation (FAO) by deacetylating liver kinase B1 and activating AMP-activated protein kinase [111]. This cascade resulted in elevated ROS levels, culminating in renal tissue apoptosis and exacerbating kidney injury. Furthermore, NADPH Oxidase 2 (NOX2) and 4 (NOX4) mediated ROS production, serving as crucial inflammatory mediators in proximal tubular cell injury [112] and necroptosis [113] following cisplatin-induced AKI. In sum, the accumulation of ROS served as both an initiator and mediator in AKI, offering novel insights for potential clinical interventions [114].

##### Inflammation and immune infiltration

**Fig. 4:**
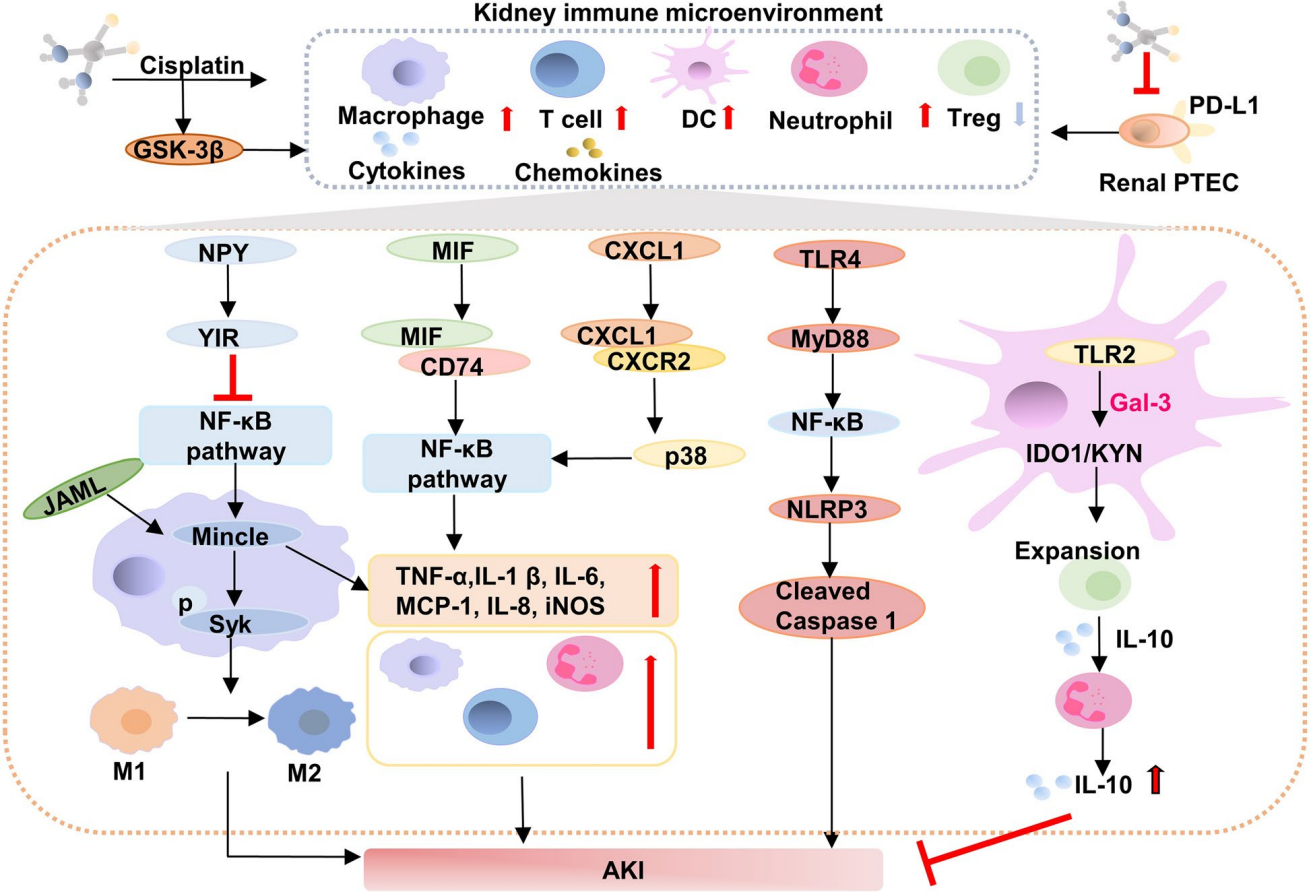
The molecular mechanism of cisplatin-induced AKI by regulating immunity and inflammation. The immune cells including macrophage, dendric cells, T cells, neutrophil, Tregs were involved in AKI. Meanwhile, the cytokines and chemokines secreted from these immune cells also contribute to AKI after administrating cisplatin. Note: AKI: Acute kidney injury; DC: Dendritic cell; Treg: Regulatory T cells; NF-κB: Nuclear factor kappa-light-chain-enhancer of activated B cells; GSK-3β: glycogen synthase kinase-3β; CXCL1: chemokine (C-X-C motif) ligand 1; CXCR2: CXC chemokine receptor 2; MyD88: Myeloid differentiation primary response 88; TLR4: Toll-like receptor 4; NLRP3: Nucleotide oligomerization domain-like receptor protein 3; IFN: interferon; IL: Interleukin; TNF-α: Tumor necrosis factor-α; MCP-1: Monocyte chemoattractant protein-1; Gal-3: Galectin 3; IDO1: Indoleamine 2,3-dioxygenase 1; KYN: Kynurenine; TLR: Toll-like receptor; iNOS: inducible nitric oxide synthase; JAML: junctional adhesion molecule-like protein; Mincle: Macrophage-inducible C-type lectin; NPY: Neuropeptide Y; Y1R: NPY receptor 1; SYK: spleen tyrosine kinase. (figure was created in BioRender. Xu, X. (2025) https://BioRender.com/f8wgo85)

Besides inducing cytotoxic effects, cisplatin also triggers an inflammatory response that contributes to nephrotoxicity and exacerbates renal tissue damage (Fig. 4**).** Cisplatin can alter the immune microenvironment of the kidneys during AKI progression [115, 116]. Hsing CH and colleagues demonstrated that cisplatin robustly activated glycogen synthase kinase-3β (GSK-3β) in vivo within the kidneys and in vitro in PTEC, leading to elevated cytokine/chemokine production, as well as the infiltration of inflammatory immune cells such as neutrophils, monocytes/macrophages, and T lymphocytes [117]. Significantly, exposure to cisplatin dose-dependently reduced the levels of renoprotective programmed death-ligand 1 (PD-L1) in primary renal PTEC, resulting in increased infiltration of CD4 + T cells and macrophages alongside reduced regulatory T cells (Treg) populations [118]. Cytokines and chemokines serve as key regulators in the development of cisplatin-induced AKI. Specifically, chemokine (C-X-C motif) ligand 1 (CXCL1)/C-X-C chemokine receptor (CXCR2) regulates inflammatory responses induced by cisplatin through the P38 and NF-κB signaling pathways both in vitro and in vivo [119]. Toll-like receptors (TLRs) also significantly contribute to AKI resulting from cisplatin exposure; for example, the nephrotoxic TLR4/NLR family pyrin domain containing-3 (NLRP3) pathway [120] and the renoprotective TLR-2 pathway [121]. Gal-3 is essential for the TLR-2-dependent activation of renal dendritic cells, stimulating the indoleamine 2, 3-dioxygenase 1 (IDO1)/kynurenine (KYN) pathway, which subsequently enhances the proliferation of immunosuppressive Tregs during kidney injury [122]. Furthermore, macrophage migration inhibitory factor (MIF) acts as an upstream pro-inflammatory cytokine, initiating the inflammatory cascade response, and activating macrophages, and T cells. In cisplatin-induced AKI, MIF instigates renal inflammation through a pathway that relies on CD74 and NF-κB signaling [123]. In recent years, increasing attention has been paid to the role of non-coding RNAs in cisplatin-induced AKI [124, 125] (Table 2). Mesenchymal stem cells (MSCs) have emerged as a promising therapeutic approach to modulate the inflammatory pathways mediated by miR-210/Serpine1 and miR-378/Fos in cisplatin-induced AKI [126]. It is proposed that cisplatin-induced nephrotoxicity results from a pro-inflammatory reaction.

**Table 2:** The regulatory mechanism of non-coding RNAs involved in cisplatin-related AKI

miRNAs	Expression after cisplatin	Models	Regulation of AKI	Target	Role	References
miR-155	up	animal and HK-2 cells	positive	TRF1 and CDK12	TECs apoptosis and DNA damage	[98]
miR-709	up	animal	positive	TFAM	oxidative stress	[110]
miR-210	-	-	-	Serpine1	inflammation	[126]
miR-378	-	-	-	Fos	inflammation	[126]
miR-483-5p	up	animal and NRK-52E cells	positive	GPX3	oxidative stress and tubular cell apoptosis	[132]
miR-186	down	animal and NRK-52E cells	negative	ZEB1	improved NRK-52E cell proliferation and protected NRK-52E cells against cisplatin-triggered apoptosis	[133]
miR-26a	down	animal and HK-2 cells	negative	TRPC6/DRP1	inhibit the mitochondrial apoptosis pathway	[134]
miR-30e-5p	down	animal and cells	positive	Galnt3	activate AMPK signaling pathway	[135]
miR-25	down	animal	positive	Ox-LDL/NOX4	inflammation and tissue damage	[136]
miR-125b	-	-		MFN1	mitochondrial fusion, apoptosis	[138]
miR-132-3p	up	-	positive	SIRT1/NF-κB signaling pathway	apoptosis and inflammatory responses	[139]
miR-199-3p	up	animal and HK-2 cells	positive	mTOR	cell apoptosis and inhibited caspase-3 activity	[140]
miR-449	up	NRK-52E cells	positive	SIRT1/p53/BAX pathway	inhibited cell viability, accelerated cell apoptosis	[141]
miR-107	up	animal and HK-2 cells	positive	RPS19	increase apoptosis	[142]
miR-34a	-	animal	-	SIRT1	oxidative stress, inflammation, and kidney injury	[143]
miR-500a-3p	up	animal and HK-2 cells	positive	MLKL pathways	inflammatory response in tubular cells and necroptosis	[148]
miR-214-3p	up	animal and TCMK-1- cells	positive	GPX4	TEC ferroptosis	[160]
circ-0114427	up	cells	positive	miR-494/ATF3/IL-6 pathway	regulates inflammatory progression	[125]
LncRNA OIP5-AS1	down	animal and HK-2 cells	negative	miR-144-5p/PKM2	renal epithelial cell apoptosis	[144]
LncRNA PRNCR1	down	animal and HK-2 cells	negative	miR-182-5p/EZH1	reduce the apoptosis of renal epithelial cells	[145]

*AKI* Acute kidney injury, *TEC* Tubule epithelial cells, *TRF1* Telomeric repeat binding factor 1, *CDK12* Cyclin-dependent kinase 12, *TFAM* Mitochondrial transcriptional factor A, *GPX3* Glutathione peroxidase 3, *ZEB1* Zinc finger E-box binding homeobox 1, *TRPC6* Transient receptor potential channel 6, *DRP1* Dynamin-related protein 1, *AMPK* Adenosine 5'-monophosphate activated protein kinase, *Galnt3* Polypeptide N-acetyl-galactosaminyltransferases 3, *Ox-LDL* Oxidized low-density lipoprotein, *NOX4* NADPH Oxidase 4, *MFN1* Mitofusin1, *SIRT1* Sirtuin1, *NF-κB* Nuclear factor kappa-light-chain-enhancer of activated B cells, *mTOR* Mechanistic target of rapamycin, *BAX* BCL-associated X protein, *RPS19* Ribosomal protein S19, *MLKL* Mixed lineage kinase domain-like protein, *GPX4* Glutathione peroxidase 4, *ATF3* Activating Transcription Factor 3, *IL* interleukin, *OIP5-AS1* Opa-interacting protein 5 antisense RNA 1, *PKM2* Pyruvate kinase M2, *PANCR1* Prostate cancer-associated non-coding RNA1, *EZH1* Enhancer of zeste homolog 1, *TCMK-1* murine renal tubular epithelial cells

##### Regulating PANoptosis and autophagy

The exposure to cisplatin leads to DNA damage, ROS generation, and inflammation, culminating in the activation of cell death pathways in tubular cells, notably the Notch signaling pathway [127]. In cisplatin-induced AKI, the predominant forms of tubular cell death are apoptosis and necroptosis. Aberrant apoptosis of renal TECs is primarily driven by endoplasmic reticulum (ER), death receptor, and mitochondrion-mediated apoptosis pathways, with the involvement of apoptotic factors such as cytochrome C (CytC) and cysteinyl aspartate-specific proteinase 3 (Caspase 3) [128, 129]. Cisplatin induces the activation of multiple mediators associated with ER stress, including glucose-regulated protein 78 (GRP78), C/EBP homologous protein (CHOP), phosphorylated c-Jun N-terminal kinase (p-JNK), protein kinase R-like ER kinase (PERK), activating transcription factor-6 (ATF-6), and inositol-requiring enzyme-1 (IRE-1) pathways. In the context of cisplatin-induced AKI, silencing of free fatty acid receptor 4 (FABP4) has been shown to reduce renal tubular cell death by mitigating ER stress [130]. Recent studies have increasingly highlighted the role of microRNAs (miRNAs) in mediating cisplatin-induced injury to renal TECs (Table 2), particularly in relation to apoptosis [98, 131–139]. Yang et al. discovered that p53 suppresses mTOR activation through the upregulation of miR-199a-3p, consequently enhancing caspase-3 activity and promoting cell apoptosis [140]. Targeting microRNAs has been identified as a promising strategy to mitigate kidney damage, with notable candidates including miR-449 [141], miR-107 [142], and miR-34a [143]. Additionally, non-coding RNAs such as long non-coding RNAs (lncRNAs) Opa interacting protein 5-antisense RNA 1 (OIP5-AS1) and Prostate Cancer Associated Non-Coding RNA 1(PRNCR1) have been implicated in AKI caused by cisplatin exposure [144, 145]. Necroptosis and necrosis are commonly observed in AKI following cisplatin administration. In kidneys treated with cisplatin, the activation of poly(ADP-ribose) polymerase 1 (PARP1) converges multiple pathways leading to cellular necrosis and inflammation, exemplified by the PARP1/TLR4/p38/Tumor Necrosis Factor-alpha (TNF-α) axis [146] and the receptor‐interacting protein kinase 1 (RIPK1)/RIPK3/mixed lineage kinase domain‐like protein (MLKL) pathway [147]. Furthermore, miR-500-3p has emerged as a pivotal regulator in cisplatin-induced necroptosis [148]. Pyroptosis, a lesser-explored pathway in cisplatin-induced AKI, involves NF-κB-mediated activation of the NLRP3/Caspase-1/Gasdermin D (GSDMD) pathway, as confirmed in the AKI model post-cisplatin treatment [149].

**Fig. 5:**
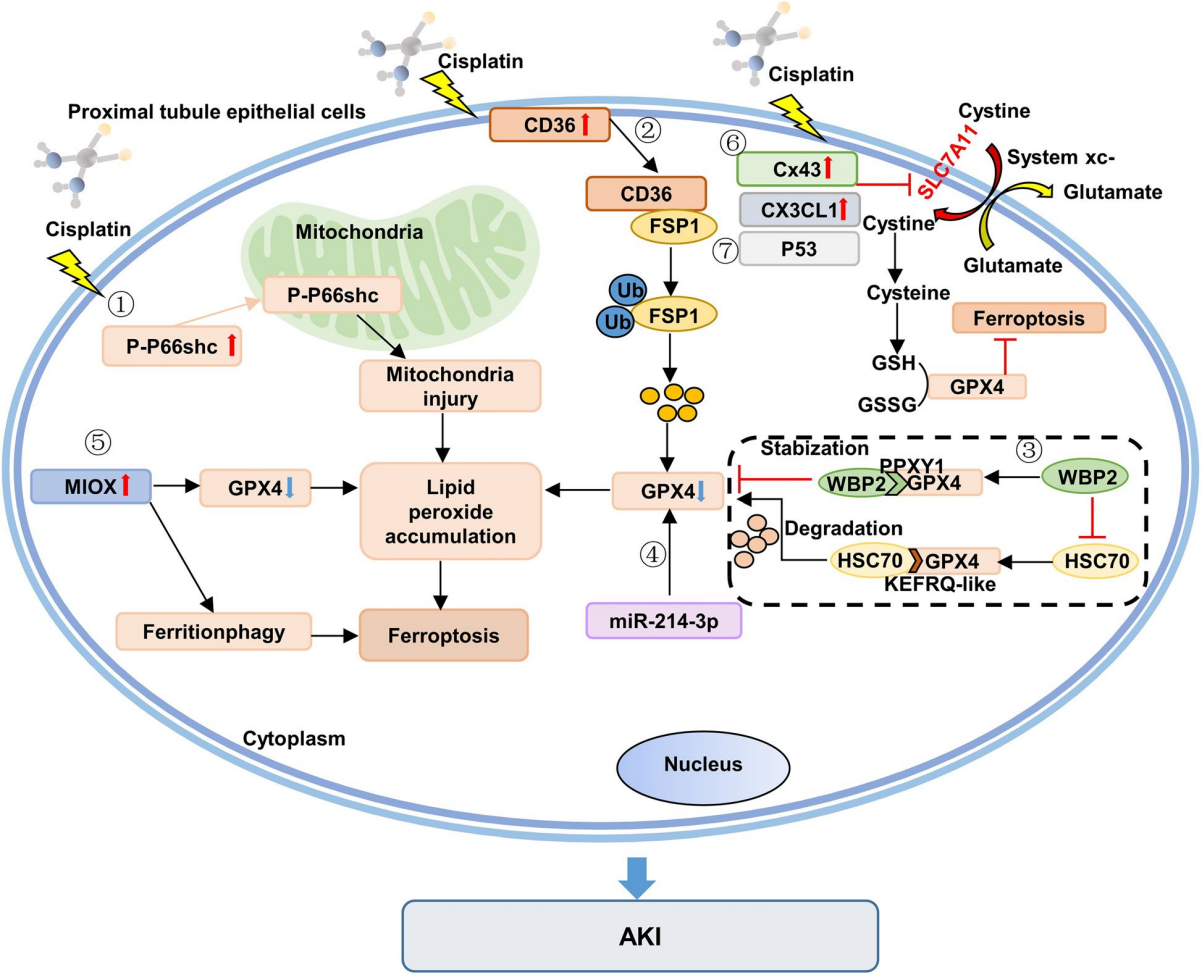
The molecular mechanism of cisplatin-induced AKI by ferroptosis. Cisplatin enters the PTECs, inactivated GPX4 and interfere with iron-dependent lipid peroxidation by several pathways. Note: AKI: Acute kidney injury; PTECs: proximal tubular epithelial cells; GPX4: glutathione peroxidase 4; MIOX: Myo-inositol oxygenase; GSH: glutathione; WBP2: WW domain binding protein-2; HSC70: Heat shock cognate protein 70; GSSG: Oxidized glutathione; FSP1: Ferroptosis suppressor protein 1; Ub: Ubiquitination; Cx43: Connexin 43; SLC7A11: Light chain subunit solute carrier family 7 member 11. (figure was created in BioRender. Xu, X. (2025) https://BioRender.com/f8wgo85)

Ferroptosis, a newly recognized form of regulated cell death observed in cisplatin-induced nephropathy, is distinguished by iron-dependent lipid peroxidation (Fig. 5) [150–156]. The disruption of the balance between ROS and antioxidants, particularly the inadequacy of glutathione (GSH), enhances ferroptosis by inhibiting glutathione peroxidase 4 (GPX4). Myo-inositol oxygenase (MIOX), an enzyme present in proximal tubules, exacerbates cellular oxidative stress in AKI. Analysis of genomic DNA epigenetics has revealed that hypomethylation of the MIOX promoter induced by cisplatin might contribute to MIOX upregulation during AKI development [157]. Cisplatin-induced ROS production intensifies MIOX overexpression, leading to lipid hydroperoxidation in both in vivo and in vitro models exposed to cisplatin [158]. These findings underscore the significant regulatory role of MIOX in cisplatin-induced AKI. Recent studies have highlighted that activation of the vitamin D receptor (VDR) offers protection against cisplatin-induced renal damage by partially inhibiting ferroptosis, which is partly mediated through the trans-regulation of GPX4 [159]. Decreased levels of miR-214-3p have been demonstrated to protect renal TECs from ferroptosis and alleviate tubular damage in cisplatin-induced AKI, as evidenced in both cellular and animal models, by upregulating GPX4 and solute carrier family 7, member 11(SLC7A11) while downregulating Acyl-CoA synthetase long-chain family member 4(ACSL4) expression [160]. These combined findings further strengthen the link between ferroptosis, and AKI induced by cisplatin.

Autophagy has been confirmed to serve a dual role in cisplatin-induced AKI [161]. Initially, following cisplatin exposure, autophagy is activated to uphold cellular homeostasis. Nonetheless, the administration of high doses (≥ 50 μM) or prolonged cisplatin treatment markedly diminishes autophagic activity [162]. The role of mitophagy in cisplatin-induced AKI has generated inconsistent perspectives across both in vitro and in vivo studies [163]. Some studies have suggested that the knockdown of phosphatase and tensin homolog (PTEN)-induced putative kinase 1 (PINK1) and Parkin RBR E3 ubiquitin -protein ligase (PRKN) worsened cellular injury and mitochondrial function, promoting a protective role against AKI [164]. Conversely, an alternative investigation put forth a contrasting perspective whereby Pink1 deficiency inhibited excessive mitophagy and dynamin 1-like (DNM1L)-mediated mitochondrial fission, potentially mitigating the impact of cisplatin-induced AKI in rats [165]. Certain signaling pathways regulate autophagy in reaction to cisplatin stimulation. Cisplatin can trigger Toll-like receptor 2 (TLR2) activation, leading to the phosphorylation of PI3K and Akt [166], or interact with heme oxygenase-1 (HO-1) pathways [121] to promote autophagy in renal tubular epithelial cells, thereby safeguarding them against cisplatin-induced AKI. Moreover, the Keap1-Nrf2 signaling pathway, known for its role in responding to oxidative stress, is implicated in the regulation of autophagy [167].

##### Other mechanisms

In addition to cell death, cell cycle regulation plays a vital role in this pathological process. Cisplatin-induced cellular damage triggers the activation of cyclin-dependent kinases 4/6 (CDK 4/6) in renal TECs, subsequently resulting in the phosphorylation of Rb1, leading to its inactivation under stress conditions. This cascade of events contributes to the dysfunction of renal TECs, cell death, and kidney injury, potentially occurring through both E2F1-dependent and -independent mechanisms [168]. Cellular senescence represents an irreversible state of cell cycle arrest characterized by changes at transcriptional, metabolic, and secretory levels, as well as alterations in cellular morphology and chromatin organization. Preliminary animal studies have highlighted the presence of cellular senescence in AKI, underscoring its significant impact on disease prognosis [169]. Yang and colleagues disclosed that the expression of SirT3, regulated by free fatty acid receptor 4 (FFAR4), has an anti-senescent influence via Gq subunit-mediated CaMKKβ/AMPK signaling cascade in cisplatin-exposed mice and TECs, underscoring the involvement of tubular FFAR4 in cellular senescence via the AMPK/SirT3 pathway [170]. Furthermore, their research team unveiled a reciprocal regulatory relationship between the aryl hydrocarbon receptor (AhR) and the methyltransferase enhancer of zeste homolog 2 (EZH2) in the kidneys of mice with cisplatin-induced AKI or in murine renal tubular epithelial TCMK-1 cells, accelerating tubular senescence in the context of cisplatin-induced AKI [171]. These innovative findings offer a fresh perspective on potential targeted therapies for AKI.

Apart from the extensively researched cisplatin, alternative chemotherapeutic treatments like carboplatin and oxaliplatin are known to induce lower levels of nephrotoxicity. Methotrexate, an antimetabolite, obstructs the metabolism of folic acid. Notably, in clinical settings, high-dose methotrexate (HDMTX) can lead to AKI in a range of 2% to 12% of patients [172]. Crystal nephropathy stands as the primary cause of nephrotoxicity induced by HDMTX. The precipitation of methotrexate and its metabolites in the renal tubules is triggered by acidic urine and a high concentration of methotrexate in these tubules. The development of intrarenal crystals can result in tubular obstruction, direct toxic injury to the renal tubular epithelium, and reduced perfusion due to vasoconstriction of the afferent arterioles, thus fostering the occurrence of AKI. Additionally, interactions between drugs can impede the excretion of methotrexate, leading to delayed clearance and subsequent nephrotoxic effects [173]. Conversely, the concurrent administration of levetiracetam and HDMTX is considered safe for adult lymphoma patients [174]. Therefore, addressing chemotherapy-related nephrotoxicity is crucial for reducing the risk of AKI.

#### Immunotherapy and acute kidney injury

**Fig. 6:**
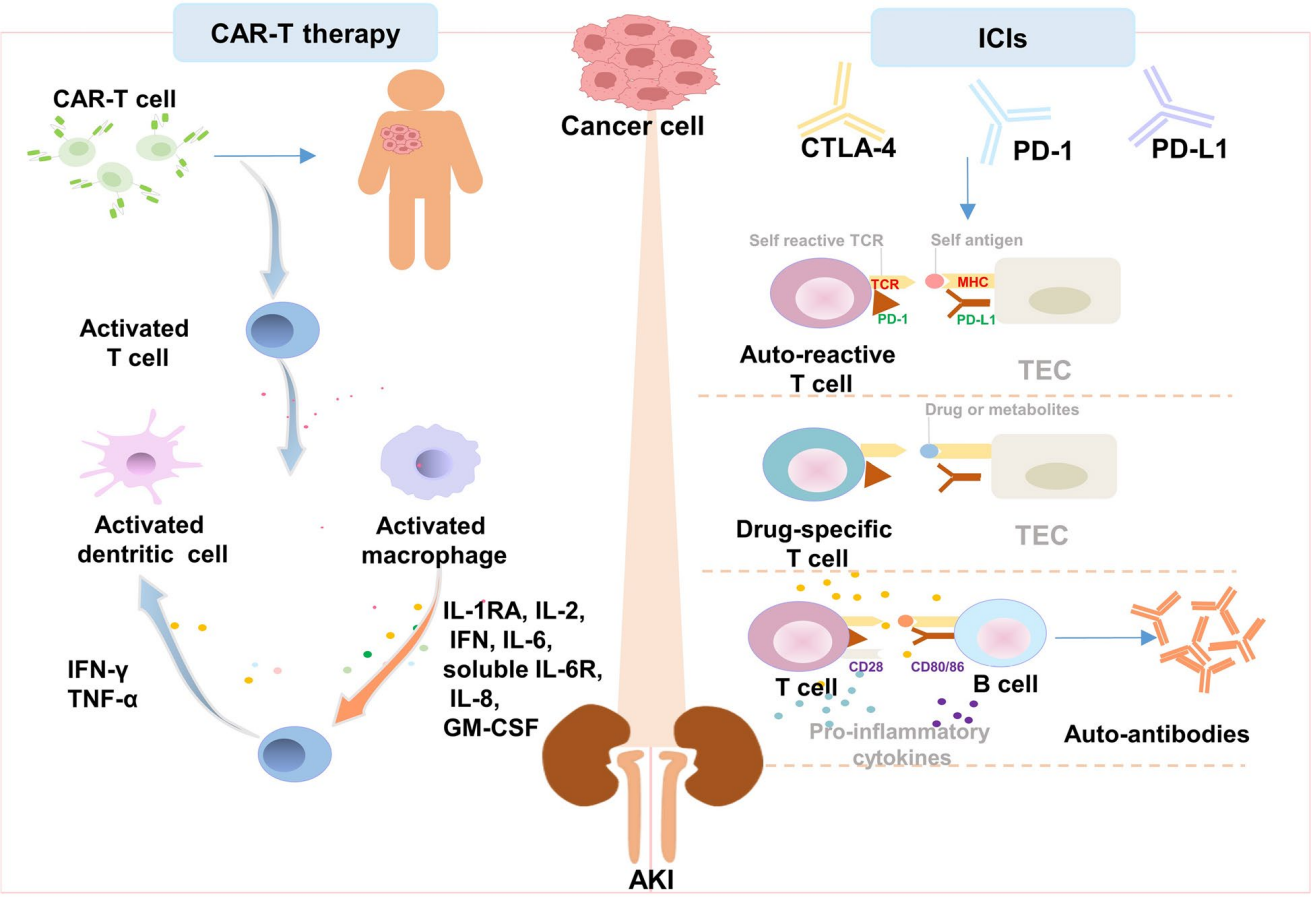
The mechanism of immunotherapy associated AKI. The left showed the Chimeric Antigen Receptor-T (CAR-T) cell therapy mediated AKI by activating T cells, macrophage, and dendric cells, then accompanied by relapsing some kinds of cytokines. The right indicated the immune checkpoint inhibitors (ICIs) resulted in AKI by loss of tolerance for self-antigen, activation of auto-reactive T cells or drug-specific T cells, producing autoantibodies. Note: AKI: Acute kidney injury; TEC: Tubular epithelial cell; CTLA-4: Cytotoxic T Lymphocyte antigen 4; PD-1: Programmed cell death protein 1; PD-L1: Programmed death- ligand 1. (figure was created in BioRender. Xu, X. (2025) https://BioRender.com/f8wgo85)

The advent of immunotherapy has brought about a revolution in the field of oncology and immune disorders. Present-day immunotherapy encompasses CAR-T cell therapy, ICIs, and other forms of immunotherapy. The mechanisms by which immune therapy may contribute to the development of AKI have been extensively explored in several studies [175–177] (Fig. 6). CAR-T cells directly engage with and eradicate cancer cells, effectively countering the immune evasion strategies utilized by malignant cells. Both CAR-T cell therapy and bispecific T cell-engaging antibodies can trigger AKI, which is frequently associated with cytokine release syndrome (CRS), TLS, sepsis, or the infiltration of specific CAR-T cells into the kidneys. In recent years, drugs in the ICIs subgroup, including anti-cytotoxic T Lymphocyte antigen 4 (CTLA-4) antibodies (ipilimumab, tremelimumab), anti-programmed cell death protein 1 (PD-1) antibodies (nivolumab, pembrolizumab), and anti-PD-L1 antibodies (atezolizumab), have been approved for clinical use. The onset of kidney involvement, a rare complication and the most delayed irAEs associated with ICIs occurs due to neonatal Fc receptor-mediated transportation and receptor-mediated endocytosis, typically manifesting after a median duration of 14 weeks following the initiation of ICIs therapy and affecting 1% to 5% of patients undergoing such treatment. Kidney biopsies frequently reveal acute tubular interstitial nephritis (ATIN) as the most common manifestation of nephrotoxicity related to immune checkpoint blockade, although glomerular diseases have also been documented. In essence, immune checkpoint inhibitors are capable of inducing AKI via several mechanisms, including the loss of tolerance to self-antigens, reactivation of drug-specific effector T cells, and the production of autoantibodies directed against renal-specific antigens [178].

##### Cytokine release syndrome

CRS is a prevalent and potentially life-threatening complication that ensues following the administration of CAR-T cells and bispecific antibodies [179]. Approximately 75% of patients undergoing CAR-T cell therapy will experience CRS as a result of CAR-T cell expansion. The incidence of severe CRS significantly increases the risk of developing AKI by 5–tenfold [180, 181]. Activated immune cells, primarily macrophages and CAR-T cells, produce substantial quantities of inflammatory cytokines, such as interleukin-1 receptor antagonist (IL-1RA), IL-2, interferon (IFN), IL-6, soluble IL-6R, IL-8, and granulocyte–macrophage colony-stimulating factor (GM-CSF). IL-6 specifically plays a critical role in CRS development. The elevated cytokine levels can induce vasodilation, reduced cardiac output, and intravascular volume depletion due to increased vascular permeability and space, resulting in diminished renal perfusion and subsequent AKI [182].

Furthermore, hemophagocytic lymphohistiocytosis (HLH) or macrophage activation syndrome (MAS) is another syndrome within the spectrum of CRS that may be associated with AKI. This syndrome is believed to be influenced by elevated levels of IL-6 and IL-10 [183]. Prerenal physiology is often triggered by cytokine-mediated vasodilation and capillary leakage, leading to a significant accumulation of fluids in the third space. The systemic toxicity that may arise from CAR-T cells has the potential to cause AKI [184]. Just as in acute cardiorenal syndrome, where kidney perfusion is reduced resulting in prerenal AKI or progression to ischemic AKI, acute cardiomyopathy due to CRS can lead to hypotension, aggravating renal hypoperfusion.

##### The aberrant activation of self-reactive T-cells and drug-specific T cells

The initiation of ICIs can result in the abnormal activation of tissue-specific self-reactive T cells, potentially disrupting or compromising immune tolerance to renal self-antigens. The activated T lymphocytes migrate to the kidney as the site of local antigen presentation, infiltrate into the parenchyma, and release cytokines, leading to immune infiltration, inflammation, and kidney injury [185, 186]. The immune profiling analysis of the kidney using imaging mass cytometry in patients with AKI-related ICIs showed an abundance of specific immune cells in the kidney tissue such as CD4 memory cells, T helper cells, and dendritic cells (DCs) [187]. The interaction between DCs and T cells, culminating in T cell activation, is a significant factor in the development of AKI. Additionally, a documented case of AKI linked to CTLA-4 haploinsufficiency, identified as acute granulomatous tubulointerstitial nephritis, led to the disruption of FOXP3 + regulatory T cells, heightened activity of effector T cells, and lymphocytic infiltration in various organs [188]. Furthermore, the expression of PD-L1 in TECs serves to shield them from aggressive autoimmunity mediated by T cells. In murine models, PD-L1 appears to be essential for the onset of AKI and immune-related AIN, given its prevalent expression in various renal pathologies not linked to ICIs therapy. Blocking antibodies against PD-L1 and PD-L2 hindered regulatory T-cells’ protective function, leading to AKI via ATN [189, 190]. Conversely, blocking the PD-1 receptor on self-reactive T cells with antibodies disrupted the PD-1/PD-L1 signaling pathway, enabling T cell proliferation, maturation, and the production of cytotoxic molecules like granzyme B (GrzB) and perforin [191]. The widespread expression of PD-L1 in renal pathologies underscores its role in the development of AKI and immune-related AIN, irrespective of ICI therapy [192]. The delayed onset of AKI following ICI administration did not align with AIN induction through the creation of an immunogenic drug metabolite [193]. Yet, no evidence has been found for the detection of ipilimumab-, pembrolizumab-, or nivolumab-specific T cells in peripheral blood.

In the majority of studies investigating ICIs-associated AKI, patients who were taking medications associated with ATIN, such as PPIs, NSAIDs, and antibiotics, were identified [191]. These drugs, by binding to tubular antigens and acting as haptens, can instigate an immune response, either directly or indirectly. Subsequently, they become embedded in the renal parenchyma, leading to tubular injury and an immunological reaction. Upon administration of ICIs, these self-reactive T cells can become prominently activated. PPIs might serve as a latent trigger for hapten-reactive T cells, which are then further stimulated by ICIs, causing a loss of tolerance [194].

##### Other mechanisms

The administration of ICIs may lead to the generation of autoantibodies that target antigens expressed by podocytes, mesangial cells, or TECs [178]. A patient with metastatic melanoma developed nephrotic syndrome and an immune-complex glomerulopathy resembling lupus following treatment with ipilimumab. This individual also generated antibodies against double-stranded DNA and anti-nuclear antigens [195]. Moreover, Bacillus Calmette–Guérin (BCG) therapy may result in adverse effects such as AIN and local BCG disease (BCGitis) or disseminated BCG disease (BCGosis), with or without granulomas [188, 196]. Tacrolimus-induced TMA -causing AKI without systemic features is a rare entity, particularly after non-renal solid organ transplantation such as heart–lung transplantation [197].

#### Targeted therapy and acute kidney injury

##### AKI induced by proteasome inhibitor

Carfilzomib (CFZ), a frequently used proteasome inhibitor for treating myelomas, has been associated with significant renal failure in patients. Efentakis et al. demonstrated that CFZ induced nephrotoxicity, triggering apoptosis, ROS generation, and inflammation pathways by activating serum/glucocorticoid-regulated kinase 1 (SGK-1) and mineralocorticoid receptors in mouse kidneys [198]. Additionally, the antioxidant system, lipid peroxidation, cytokine activation (IL-1β, IL-6, TNF-α), caspase-3, and Nrf2 downregulation play key roles in CFZ-induced renal impairment [199]. Notably, metabolomics studies have shown that CFZ causes the retention and dysfunction of metabolites, leading to local kidney injury by inhibiting nitric oxide (NO) production, resulting in elevated kidney resistance, increased oxidative stress, and modulation of inflammation [200].

##### AKI induced by Tyrosine kinase inhibitors (TKIs) and other inhibitors

TKIs such as vandetanib have been found to inhibit a number of human renal transporters, notably MATE-1 and MATE-2, essential for removing a variety of toxins and medications [201]. Agents that target MATE-1 and MATE-2 can impede the apical membrane of tubular cells, potentially leading to elevated drug concentrations within renal cells and exacerbating the renal toxic effects of certain drugs, particularly cisplatin [202]. AKI has primarily been associated with v-Raf murine sarcoma viral oncogene homolog B(BRAF) inhibitors due to instances of ATN, rhabdomyolysis, and TLS [203]. Epidermal Growth Factor Receptor (EGFR) inhibitors consist of three small-molecule TKIs (erlotinib, gefitinib, and afatinib) and two monoclonal antibodies (cetuximab and panitumumab). Each of these agents has been linked to the occurrence of AKI. EGFR, predominantly expressed in the distal and collecting tubules, plays a vital role in maintaining tubular integrity. Upon ATN, EGFR activation stimulates the growth and regeneration of TECs. Anti-EGFR medications may serve as a “second hit” in the development of AKI in individuals predisposed to renal dysfunction. AKI cases, including ATN, TLS, and ATIN, have been observed in patients receiving BRAF inhibitors. The likelihood of AKI was higher with concurrent use of trametinib and dabrafenib. Anaplastic lymphoma kinase(ALK) inhibitors have been linked to the development of peripheral edema and, in rare cases, electrolyte disorders and kidney failure [204]. In individuals undergoing anti-vascular endothelial growth factor (VEGF) therapy, TMA is the most frequently documented histopathological manifestation associated with AKI [205].

#### Radiotherapy and radiographic contrast agents-induced acute kidney injury

The kidneys are the most radiosensitive abdominal organ, yet the molecular pathomechanisms underlying radiotherapy nephropathy (RN) remain incompletely understood. Consequently, managing radiation exposure and contrast-induced AKI remains an ongoing challenge. Programmed cell death 4 (PDCD4) may play a role in radiotherapy-induced AKI in rectal cancer by upregulating FGR expression, activating the NF-κB signaling pathway, and triggering an oxidative stress response [206]. In radiation-induced kidney injury associated with cervical cancer, PARP1 interacts with X-ray repair cross-complementing 1 (XRCC1) to form a complex, which subsequently induces DNA damage and oxidative stress response in renal tubular cells [207]. Moreover, insulin-like growth factor-1 (IGF-1) and calcitonin gene-related peptide (CGRP) are involved in the development of radiotherapy nephropathy [208]. The pathogenesis of contrast-induced AKI may be related to the direct cytotoxicity of iodine contrast medium, medullary hypoxia, and ischemia, as well as oxidative stress. Contrast agents activate the classical Nlrp3 inflammasome in macrophages, leading to AKI through the regulation of inflammation. This process requires the involvement of IL-1, leukocyte recruitment, dipeptidase-1 (DPEP-1), and resident renal phagocytes. Upon recruitment of monocytes and macrophages to the kidneys, they interact directly with tubular cells to process contrast taken up from the urine. DPEP-1, an enzyme found on the brush border, plays a crucial role in the tubular reabsorption of contrast. The NLRP3 inflammasome is implicated in contrast-induced AKI by modulating the apoptotic pathway [209, 210]. Recent renal proteomics analysis has identified 16 candidate proteins that could reveal novel mechanisms in the pathogenesis of contrast-induced AKI [211], offering new insights for potential therapeutic targets in the management of radiotherapy or contrast media-induced AKI.

### Inflammatory response, oxidative stress, and tubular injury

#### Inflammatory response in pathogenesis of acute kidney injury

Historically, kidney dysfunction was primarily attributed to hemodynamic disturbances. Nevertheless, recent research has highlighted the intricate relationship between AKI, inflammation, and the immune system. The REALAKI study demonstrated that early-onset AKI is linked to the initial inflammatory response and injury-induced immunosuppression observed at the end of the first week following severe injuries like sepsis, trauma, surgery, and burns [212]. Injured renal cells release signals recruiting lymphocytes, which either perpetuate or inhibit renal injury. Both intrarenal and systemic inflammation are linked to AKI, implicating innate and adaptive immune systems in its pathogenesis. The development and progression of AKI are notably influenced by intricate intrarenal inflammatory pathways, which are propelled by both lymphocytes and innate immune cells [213]. Innate immune cells, such as granulocytes and macrophages, can trigger AKI by secreting pro-inflammatory mediators [214]. These cells can cause direct renal damage or indirectly through the recruitment of adaptive immune cells. Evidence from studies in both humans and mice has indicated that the complement system is activated during AKI. CD4 + and CD8 + T cells, B cells, and neutrophils are probably implicated in the development and progression of AKI. In contrast, regulatory T cells, double-negative T cells, and type 2 innate lymphoid cells appear to exert protective effects. Some other immune cells, such as macrophages and natural killer T cells, may exhibit both harmful and protective effects, contingent upon their specific subtype and/or the phase of AKI. The immune system not only contributes to the injury and repair processes during AKI but also mediates AKI-induced dysfunction in distant organs. Macrophages, as the most abundant immune cells residing in the kidney, can differentiate into pro-inflammatory and anti-inflammatory phenotypes. They dynamically influence the progression of AKI and the subsequent repair processes while also helping to maintain tissue homeostasis [215, 216]. Macrophages influence the local inflammatory microenvironment by secreting extracellular vesicles (EVs) that carry pro-inflammatory or anti-inflammatory signaling molecules, thereby modulating tissue injury and repair. In rhabdomyolysis-induced AKI, necrotic muscle cells release substances that can activate platelets that could raise the production of macrophage extracellular traps (METs) by enhancing histone citrullination and intracellular ROS generation [217]. The primary signaling pathways involved in macrophage-mediated pro-inflammatory responses in AKI encompass the Notch, NF-κB, PI3K-AKT, JAK-STAT, and necroptosis pathways. Among these, Saa3hiCcl2hi monocyte-derived infiltrating macrophages have emerged as a pivotal effector subset, driving inflammation and directly interacting with renal cells [218]. During AKI, TECs acutely secrete IL-34, which activates resident macrophages and exacerbates tubular injury. T cells significantly influence the transition from AKI to CKD through their roles in injury and repair mechanisms. Importantly, T cells also participate in distant organ crosstalk during AKI, which affects overall outcomes. After AKI, TECs predominantly release signals that induce a T helper 1 (TH1) response, and renal myeloid cells polarize toward an IL-17-producing T helper (TH17) phenotype [219]. However, the role of T helper 2 (TH2) cells during AKI is still not well understood. Following renal injury, Tregs mitigate inflammation through various mechanisms. Gamma delta T cells (γδT cells) play a role in modulating the progression of kidney injury, but they are not indispensable for the development of AKI. IRI robustly recruits neutrophils and monocytes, leading to AKI. Dipeptidase-1 (DPEP1), a key neutrophil adhesion receptor, is highly expressed in renal proximal tubular cells and peritubular capillaries. DPEP1, along with CD44 and Intercellular adhesion molecule-1 (ICAM-1), plays a significant role in the recruitment of monocytes/macrophages to the kidney after IRI [220].

Damage-associated molecular patterns (DAMPs) have been recognized as substantial contributors to the development of AKI. Serum peroxiredoxin 1 (Prdx1) is a novel DAMP that mechanistically contributes to renal injury through activation of the Mincle/Syk/NF-κB signaling axis, thereby driving pro-inflammatory cascades [221]. In systemic inflammatory conditions induced by sepsis and cirrhosis, PAMPs and DAMPs potently activate innate immunity [222]. This immunostimulatory process engages multiple physiological systems, including complement activation, coagulation cascade initiation, and endothelial cell activation. The unabated inflammatory response mediated by PAMPs/DAMPs induces endothelial barrier dysfunction across vascular beds and parenchymal organs following regulated cell death (RCD). Subsequently, unregulated inflammatory mediators enter systemic circulation and exert detrimental effects on renal homeostasis, resulting in disruption of regional hemodynamic stability, impairment of renal vascular endothelial integrity, and mitochondrial dysfunction. These pathophysiological alterations manifest clinically as deteriorating renal function, biochemically characterized by elevated serum creatinine and urea nitrogen concentrations.

#### Oxidative stress and tubular injury

The pathological mechanisms underlying AKI are complex, with oxidative stress emerging as a major contributor. Like drug-induced and contrast-induced AKI, the kidney is highly susceptible to IRI under various clinical conditions, including hypotension, sepsis, and surgical procedures such as partial nephrectomy and kidney transplantation. IRI disrupts the cellular redox balance, triggering excessive production of ROS in the kidneys upon reperfusion. This triggers a series of events typical of IRI-induced AKI, such as mitochondrial damage, energy depletion, tubular apoptosis, and necrosis. Mitochondrial DNA (mtDNA) damage is recognized as a characteristic feature of AKI, with mtDNA depletion documented in both preclinical models and human patients with AKI. Mitochondrial ROS (mtROS) can worsen renal injury by suppressing mitochondrial transcription factor A (TFAM)-mediated maintenance of mtDNA. This suppression results in decreased mitochondrial energy metabolism and increased cytokine release. These findings suggest that addressing TFAM deficiencies could be a potential therapeutic approach for renal repair following IRI-AKI [223]. Oxidative stress is a key pathogenic mechanism of AKI, hence oxidative stress-related pathways are the key regulator in AKI, including the NOXs pathway, AMPK/PKC pathway, Nrf2/HO-1 pathway, Nrf2/SIRT3/SOD2 pathway, SIRT1/PGC-1α/FoxO1 pathway.

Previous studies uncovered accumulated signaling pathways involved in the occurrence and development of AKI [224], including inflammation, oxidative stress, and regulated cell death-related pathway. Oxidative stress and inflammation are intricately interconnected, forming a vicious cycle where oxidative stress triggers inflammation via various molecular mechanisms, including the NF-κB signaling pathway [225]. It is widely recognized that AKI is not always characterized by the loss of kidney epithelial cells, although cell death is common in severe cases of AKI. Importantly, there are intricate connections between different forms of regulated cell death, and even between regulated necrosis and inflammatory processes. Pathways of regulated cell death, including apoptosis and necrosis, have been identified as key events in the pathogenesis of AKI [226]. Apoptosis pathways mediated by mitochondrial autophagy, Bax/Bcl-2/caspase3, 9 pathway, MAPK pathway, PI3k/Akt/mTOR/Nrf2 pathway, and ER stress were the dominant contributors in various AKI. Notably, regulated necrosis pathways, such as ferroptosis, necroptosis, and proptosis, and mitochondrial permeability transition-mediated regulated necrosis, can directly induce AKI or contribute to its development by recruiting immune cells and triggering inflammatory responses [227]. Gasdermins E (GSDME)-mediated pyroptosis, mitochondrial damage, and inflammation played an important role in renal IRI, such as CHOP/Caspase3/GSDME mechanistic axis [228]. F-Box and WD Repeat Domain Containing 7 (FBXW7) exacerbates IR-AKI by promoting ferroptosis through downregulation of GPX4 expression [229]. mmu-lncRNA129814/hsa-lncRNA582795/miRNA-494-5p/IL-1α axis was found to modulate the progression of ischemic AKI [230]. In addition to the commonly employed pathways mentioned above, several emerging pathways have also been reported in the context of AKI. For example, the OPG/RANKL/RANK/TLR4 signaling cascade is implicated in the pathogenesis of sepsis-associated AKI [231]. The hypoxia-regulated pathway is also a significant contributor to the development of AKI. Hypoxic AKI is frequently observed in widely used warm ischemia–reperfusion (WIR) models, typically characterized by severe proximal tubular injury and accompanied by significant inflammation [232]. S100A16 worsens AKI by enhancing the ubiquitylation and degradation of glycogen synthase kinase 3β (GSK3β) and casein kinase 1α (CK1α) via the activation of HMG-CoA reductase degradation protein 1 (HRD1). TFAP2B, functioning as the transcription factor for S100A16, highlights the role of HIF-1α in regulating HRD1 transcription within the S100A16-HRD1-GSK3β/CK1α pathway during renal hypoxia injury [69]. FHL2 may be essential for protecting against ischemic AKI by enhancing the activation of HIF-1 and β-catenin signaling through its interactions with various protein partners [233]. The TWEAK/Fn14 axis is involved in the pathogenesis of rhabdomyolysis-induced AKI and the progression from AKI to CKD [71]. Collectively, a better comprehensive understanding of the pathophysiology of AKI is beneficial to establish the risk stratification for recognizing the susceptible subpopulation to AKI.

## Diagnosis of acute kidney injury

### Clinical criteria and biomarkers

#### Clinical criteria for diagnosis of acute kidney injury

AKI was classified according to three criteria sets (Table 3): RIFLE, AKIN, and KDIGO. For each definition, only the serum creatinine criteria were utilized, while urine output criteria were not considered. More importantly, early identification of AKI utilized the diagnosis of AKI and precise intervention. The AKI-Pro score, which encompasses factors such as female, baseline eGFR, aortic surgery, modified furosemide responsiveness index (mFRI), SOFA (Sequential Organ Failure Assessment) score, and AKI stage, represents a novel clinical tool. It offers valuable guidance for identifying early AKI patients who are at a heightened risk of experiencing AKI progression or succumbing to death [234].

**Table 3:** AKI criteria according to RIFIE, AKIN, KDIGO

AKI staging	Urine output	Scr		
		RIFLE	AKIN	KDIGO
1	Less than 0.5 ml/kg per hour for over 6 h	Serum Cr rising to 1.5 times the baseline level, GFR decreasing by 25%, or urine output less than 0.5 mL/kg/h for 6 h	An increase in creatinine of ≥ 50%, with an additional 0.3-mg/dl increase for stage 1, or AKI diagnosed within a 48-h period, characterized by an absolute increase in creatinine of 0.3 mg/dl	An increase in serum creatinine of 0.3 mg/dL or more (26.5 μmol/L or more) within 48 h, or an increase to 1.5 times or more than the baseline value from the prior 7 days
2	Less than 0.5 ml/kg per hour for more than 12 h	Injury: Cr ↑ of 2 × baseline, GFR ↓ of 50%, or urine output < 0.5 mL/kg/h for 12 h	Increase in creatinine of ≥ 100%	An elevation in serum creatinine to 2.0–2.9 times or higher than the baseline value from the preceding 7 days
3	Less than 0.3 ml/kg per hour for more than 24 h or anuria for 12 h	Failure: Cr ↑ of 3 × baseline, GFR ↓ of 75%, Cr ≥ 4.0, or urine output < 0.5 mL/kg/h for 12 h	Increase in creatinine of ≥ 200%	Increase in serum creatinine by 4.0 mg/dL or more (353.6 μmol/L or more); or Increase in serum creatinine to 3.0 times or more than the baseline; or initation of RRT; or eGFR ≤ 35 ml/min per 1.73 m^2^ (if age < 18 yr)
4		Loss: Kidney function loss lasting more than 4 weeks		
5		ESRD: Loss of kidney function for over 3 months		

*RIFLE* Risk, Injury, Failure, Loss, and End-stage renal disease, *AKIN* Acute Kidney Injury Network, *KDIGO* Kidney Disease: Improving Global Outcomes, *Scr* serum creatinine, *eGFR* Estimating Glomerular Filtration Rate

#### Biomarkers-guided diagnosis for acute kidney injury

The biomarker related to AKI consisted of stress biomarkers, functional biomarkers, damage biomarkers, and cell cycle arrest biomarkers. Traditionally methods using only serum creatinine and urine output defined AKI, limited by certain factors. Novel biomarker-based diagnostics displayed superiority over the traditional methods(Table 4). Currently, cystatin C (CysC), serum creatinine(Scr), blood urea nitrogen (BUN), and urinary output are commonly employed as functional biomarkers for predicting AKI. In particular, damage biomarkers mainly included kidney injury molecule-1 (KIM-1) and neutrophil gelatinase-associated lipocalin (NGAL). The US Food and Drug Administration (FDA) has granted approval to KIM-1 as a biomarker for nephrotoxicity associated with several currently used drugs. Additionally, other biomarkers that signal nephrotoxic damage encompass alanine aminopeptidase (AAP), glutathione s-transferase (GST), and gamma-glutamyl transpeptidase (GGT). Urinary dickkopf-3 (DKK3), a glycoprotein produced by TECs in response to stress and known for its profibrotic effects, has demonstrated its potential in predicting postoperative AKI. It also offers valuable insights into persistent tubulointerstitial fibrosis and transient eGFR decline. Liver-type Fatty Acid-Binding Protein (L-FABP) primarily synthesized from the liver and also produced in other organs such as the kidney, can measured in urine to forecast the occurrence of AKI in patients who have undergone cardiac surgery or those in critical condition, with an apparent advantage over NGAL. The lysosomal enzyme N-acetyl-b-D-glucosaminidase (NAG) and the cytosolic protein lactate dehydrogenase (LDH) are also notable biomarkers. Given that G1 cell cycle arrest triggered by cellular stress is one of the early occurrences in AKI, urinary insulin-like growth factor-binding protein 7 (IGFBP7) and metalloproteinase inhibitor 2 (TIMP2) can be detected at the onset of AKI development. Previous research based on the physiology of AKI expanded mitochondrial-related biomarkers for predicting renal dysfunction [235]. Pannexin 1 (PANX1), a key regulator of ATP release, holds promise as a biomarker associated with mitochondrial dysfunction for diagnosing IRI-AKI [236]. Urinary full-length ATP synthase subunit beta (ATPSb) emerges as a highly sensitive and specific biomarker reflecting renal mitochondrial dysfunction in IRI-AKI [237]. Higher levels of mitochondrial DNA in urine can act as a proxy biomarker for kidney injury [238]. The kidney in older individuals is particularly susceptible to acute injury stemming from ischemia–reperfusion, toxic drugs, modified matrix proteins, systemic hemodynamics, and other factors. Non-coding RNA and microRNAs (miRNAs) are vital for maintaining renal homeostasis, and alterations in their expression patterns can serve as valuable indicators for AKI [239]. Additionally, some novel biomarkers for managing AKI are oncoming, such as ferroptosis-related gene *Gclc* [240], inflammatory biomarkers CXCL1 and TNFRSF12A [241], kidney-specific cfDNA methylation markers [242]. Higher levels of sTLR2 in the early stages of sepsis may emerge as a potential new indicator for predicting sepsis-associated AKI [243]. Plasma soluble urokinase plasminogen activator receptor (suPAR) and Scr are capable of specifically distinguishing critically ill children who are at a high risk of developing non-septic AKI and sepsis-associated AKI [244]. Among patients with moderate to severe AKI, urinary C–C motif chemokine ligand 14 (CCL14) demonstrated remarkable efficacy in predicting the persistence of AKI [245]. Urinary vanin-1 serves as an exceptional minimally invasive biomarker for the early prediction of cisplatin-induced AKI [246]. The levels of urine complement C3 and vitamin D-binding protein may serve as promising early indicators of adverse outcomes in patients who experience AKI following cardiac surgery [247]. There was also some biomarkers based on clinical laboratory tests for predicting AKI, like systemic immune-inflammation index (SII) [248], and high-sensitivity C-reactive protein (hsCRP) [249].

**Table 4:** The advantages of biomarker-based diagnostics

	Biomarker-based diagnostics	Sample type	Time	Advantages	Disadvantage
Injury markers	NGAL	urine	2-4 h after injury	early diagnosis; differentiating prerenal AKI from renal AKI	May be elevated in the settings of sepsis, chronic kidney disease, and urinary tract infection
	KIM-1	urine	12–24 h after injury		May be elevated in the settings of chronic proteinuria and inflammatory diseases
	GGT,GST,AAP	urine	-		Increased in urinary tract infections, cardiovascular disease, and cerebrovascular events
	L-FABP	urine and plasma		early diagnosis	Linked to anemia in individuals without diabetes; in postcardiac surgery and ICU settings; High cost and poor availability
	DKK3	urine		predict postoperative AKI	
Stress marker	TIMP2 and IGFBP7	urine	Occurring as soon as 4 h, yet generally within 12 h	within 72 h after ICU admission; predicting the development of moderate or severe AKI	
Functional marker	Cystatin C	plasma	Within 12 to 24 h following the injury	early diagnosis	Influenced by a variety of factors
Traditional marker	SCr	serum			Inaccurate in patients with low muscle mass or with fluid overload; low sensitivity and specifity in early stage of AKI
	urine output	urine		cheap	low sensitivity and specifity in early stage of AKI; influenced by infusion or diuretic

*NGAL* neutrophil gelatinase-associated lipocalin, *KIM-1* Kidney Injury Molecule-1, *GGT* gamma-glutamyl transpeptidase, *GST* Glutathione S-transferase, *AAP* alanine aminopeptidase, *DKK3* dickkopf-3, *L-FABP* Liver-type Fatty Acid-Binding Protein, *TIMP2* metalloproteinase inhibitor 2, *IGFBP7* insulin-like growth factor-binding protein 7

### Role of imaging studies

Imaging modalities, including ultrasonography, X-ray, magnetic resonance imaging (MRI), or computed tomography (CT) often yield characteristic UTO images that support the diagnosis of AKI, particularly in cases of post-renal AKI, under most circumstances. Recently, renal contrast-enhanced ultrasonography (CEUS) and multiparametric MRI (mpMRI) seem to be the most potential imaging methods for evaluating renal microcirculation and oxygenation involved in AKI. Ultrasound molecular imaging, utilizing targeted microbubbles (TM) loaded with vascular cell adhesion molecule-1(VCAM-1) polypeptide, possesses the capability to precisely assess alterations in renal microcirculatory perfusion and inflammatory response. This innovative approach may emerge as a highly potential approach for the early detection of AKI [250]. In recent years, the application of renal MRI in the assessment of AKI has been on the rise, with its utilization in preclinical studies surpassing that in clinical studies. CF@P possesses antioxidant/anti-inflammatory properties by inhibiting pyruvate dehydrogenase kinase 4 (PDK4) and exhibited promising capabilities in T1-MRI and photoacoustic imaging for AKI management [251]. Given contrast-enhanced MRI (CE-MRI) of AKI significantly restricted by the inadequate targeting ability and potential toxicity of existing contrast agents, bovine serum albumin@polydopamine@Fe (BSA@PDA@Fe, BPFe) nanoprobe with self-purification capabilities and renal tubule-targeting properties for targeted CE-MRI of AKI [40]. BPFe is also employed for CE-MR angiography to visualize fine vessel structures. To position renal MRI as a vital tool for clinical research, the integration of multiparametric MRI (mpMRI) is indispensable [252], mpMRI provides comprehensive information on renal vasculature and function, including arterial spin labeling and intravoxel incoherent motion, as well as tissue oxygenation through blood oxygen level-dependent imaging. Moreover, it offers insights into tissue injury and fibrosis via diffusion tensor imaging, diffusion kurtosis imaging, T1 and T2 mapping, and quantitative susceptibility mapping [253]. When compared to mpMRI, amine-CEST has the potential to achieve the highest accuracy in diagnosing IRI-AKI [254]. PET/^19^F MRI dual-modal imaging probes have been developed for monitoring ferroptosis and identifying the onset of cisplatin-induced AKI a minimum of 24 h earlier than conventional clinical or preclinical assays [255]. A tailored photoacoustic (PA) imaging probe (AB-DiOH) has also been created, which exhibits highly specific and sensitive reversible responses to hypochlorite (ClO-) and glutathione (GSH) [256]. This innovative probe allows for continuous, non-invasive monitoring of AKI through PA imaging, demonstrating superior detection sensitivity compared to traditional blood tests. However, there is a notable lack of clinical studies utilizing MRI to assess the degree and timing of renal recovery, and no clinical research has yet been carried out to assess the response to interventions in AKI using MRI.

### The clinical pathology of acute kidney injury

The fundamental histopathological hallmarks of AKI are characterized by the loss of the epithelial phenotype in PTECs, which is manifested by the detachment of the brush border, the flattening and localized shedding of TECs, infiltration of inflammatory cells, and the formation of casts that are rich in Tamm-Horsfall protein [257]. The damaged TECs exhibit significant cellular adaptability. In the initial phase of AKI, some TECs are damaged and undergo cell death, while the surviving TECs initiate an adaptive repair process. ATN is the most common event. Collapsing glomerulopathy is the most frequent glomerular injury and is strongly linked to apolipoprotein L1 genotypes.

### Differential diagnosis from chronic kidney diseases

Up to now, AKI and CKD have been clearly defined and categorized. This has in turn spurred enhanced research endeavors and given rise to more effective management strategies and recommendations. Firstly, medical history is a key point for differential diagnosis between AKI and CKD. Kidney injury or decline in kidney function lasting for at least 3 months is regarded as CKD. It’s important to urgently identify and timely intervention of AKI to prevent the transition from AKI to CKD. Secondly, the clinical phenotypes of the two renal impairments were distinct. Diagnosis from the clinical laboratory based on the KIDGO criteria showed the superior guideline to distinguish AKI from CKD, including functional parameters and structure change. The diagnostic criteria for GFR of below 60 mL/min per 1.73 m^2^ and an albumin-creatinine ratio (ACR) at or above 30 mg/g were maintained. However, the inclusion of the phrase “with health implications” underscores the idea that while numerous kidney structural or functional abnormalities may exist, not all of them necessarily impact an individual's health. Thirdly, imaging diagnosis usually showed a smaller volume of kidney or other abnormalities of structure in CKD cases compared to AKI with normal kidney. Besides, serum O-sulfotyrosine levels enhanced the ability to distinguish AKI from CKD [258]. Collectively, although more efforts have standardized definitions and classification systems for AKI and CKD, recognition and awareness of both disorders facilitated timely interventions.

## Therapeutic interventions

AKI is increasingly emerging as a significant issue for patients, primarily attributed to the extensive employment of nephrotoxic agents in healthcare settings. Currently, the early diagnosis of AKI remains a formidable challenge, and the existing therapeutic drugs are far from sufficient to meet the clinical demand. Medical management strategies for individuals with AKI typically encompass renal replacement therapy (RRT) and nutritional support. No definitive treatment has been established, even though various pharmacologic therapies including diuretics, vasoactive drugs, and growth factors have been documented in animal models or initial clinical trials [259, 260]. Human Urine-Derived Stem Cells are protected from PTC injury and impeded macrophage polarization, as well as the secretion of pro-inflammatory interleukins, a novel potential cell therapy in AKI [261]. Macrophage autophagy safeguards against AKI by curbing renal inflammation via the MARCHF1- and MARCHF8-mediated degradation of T cell-interacting, activating receptors on myeloid cells 1(TARM1) [262]. Given the current situation, the discovery of novel therapeutic targets for AKI is of utmost urgency.

### Supportive care strategies

Prerenal causes, originating from hypovolemia and decreased kidney blood flow, often respond well to fluid administration. Fluids are essential for maintaining kidney perfusion and compensating for impaired autoregulation mechanisms. Additionally, in various other scenarios, such as before surgery, fluids can be beneficial in preventing postoperative ATN. In the context of renal toxicity, particular attention must be paid to medications to prevent underdosing or adverse effects. Any drugs that may cause harm should be discontinued, and dose adjustments should be made based on renal function. For patients with AKI, it is crucial to promptly diagnose and treat associated complications such as metabolic acidosis, hyperkalemia, anemia, and fluid overload. Additionally, it is advisable to initiate stress-ulcer prophylaxis and take measures to prevent infections during the course of AKI. Fluid management is a crucial element for preventing and managing AKI. Traditionally, the focus has been on fluid resuscitation to ensure adequate renal perfusion. However, emerging evidence now cautions against the over-administration of fluids, considering that patients with AKI are highly vulnerable to volume overload [263]. In a cross-institutional feasibility trial conducted across seven ICUs in Europe and Australia, critically unwell individuals with AKI who were subjected to a conservative fluid management protocol experienced a lower overall fluid accumulation and fewer complications compared to those receiving usual care [264]. Of note, a more personalized approach to resuscitation is now preferred, taking into account the extent of hypovolemia. The volume of fluids administered should be precisely calculated to ensure adequate perfusion and oxygen delivery to the kidneys and other vital organs. Point-of-care ultrasound (POCUS) has emerged as a mainstream diagnostic tool for volume assessment, providing valuable insights into hemodynamics and organ congestion. The selection of fluid for resuscitation should be based on a comprehensive evaluation of the patient's volume status, electrolyte levels, the underlying cause of hypovolemia, comorbidities, and numerous other factors.

### Pharmacological treatments

Therapies such as blood pressure control, glycaemic control (for diabetic patients), renin-angiotensin inhibition, and statins may be crucial in improving long-term cardiovascular and renal outcomes following AKI. Vasopressors represent another major category of treatment employed to elevate blood pressure. Moreover, timely administration of antibiotics in septic patients is crucial. Hypoxia-inducible factor (HIF) is reported to shield the kidney from acute ischemic harm and a novel HIF stabilizer, FG4592 (Roxadustat), has been introduced into clinical practice as an anti-anemia medication [265]. A newly synthesized covalent antagonist, H151, which targets both human and murine STING, has demonstrated protective effects against renal injury in cisplatin-induced AKI possibly achieved through mechanisms involving the amelioration of inflammation and mitochondrial dysfunction [266]. There were some clinical trials involving alleviating AKI [267]. Quinolinate phosphoribosyltransferase (QPRT), a key enzyme in de novo biosynthesis, helps to maintain renal NAD + levels and confers resistance to AKI [268]. Ilofotase alfa, a recombinant human alkaline phosphatase, has been demonstrated to provide renal protection. In patients with sepsis-associated AKI, it has been demonstrated to enhance survival rates and markedly decrease the occurrence of major adverse kidney events by 90 days (MAKE90) [269]. The oral administration of an FAAH inhibitor has been shown to effectively mitigate cisplatin-induced nephrotoxicity. Specifically, it can significantly reduce the DNA damage response, tubular injuries, and overall kidney dysfunction caused by cisplatin. The inactivation of FAAH thus emerges as a highly potential approach for the prevention of nephrotoxicity associated with cisplatin treatment [270]. Cardiac surgery-associated AKI (CSA-AKI) is a severe complication in patients who undergo cardiac surgery involving cardiopulmonary bypass (CPB), which is associated with increased postoperative adverse events and mortality rates. The VERTIGO trial aims to delineate the efficacy and safety profile of empagliflozin in the prevention of CSA-AKI [271].

### Novel therapeutic approaches

Though medical research has made headway, the creation of new treatment methods for AKI is still in its nascent stages. These cutting-edge therapies have the potential to greatly enhance patient outcomes by focusing on the root causes and underlying mechanisms of AKI. A specialized photoacoustic (PA) imaging probe, AB-DiOH, exhibits high specificity and sensitivity in reversibly responding to ClO-/GSH [256]. This probe is employed to identify nephroprotective agents within natural products. Additionally, it has been discovered that the oral intake of astragalin may hold promise as a new therapeutic agent for the treatment of AKI through the inhibition of oxidative stress, ferroptosis, and cuproptosis. Berberine exerts a protective effect against IRI-induced AKI by inhibiting the intestinal biological barrier of Proteobacteria, reducing lipopolysaccharide (LPS) production, and eliciting an anti-inflammatory response via the regulation of the Sirt1-NF-κB-TLR4 pathway [272]. Dihydroartemisinin (DHA) has demonstrated its protective effect against AKI through the alleviation of oxidative stress and the reduction of inflammatory damage. These beneficial actions may be closely linked to its capacity to activate the Nrf2 pathway and modulate macrophage polarization [273]. Cullin 4B (CUL4B) exerts a kidney-protective role against AKI by restraining p53/PAI-1 signaling [274].

Therapeutic targeting of mitochondria is an emerging approach for AKI, including targeting mitochondrial energy metabolism, mitophagy, reducing ROS levels, disrupted mitochondrial fusion and fission. Mitochondrial energy metabolism involves several key processes, including pyruvate oxidation, FAO, the citric acid cycle, and the five complexes of oxidative phosphorylation. Mitochondrial damage can persist long after ischemia, perpetuating chronic inflammasome activation through the release of noxious molecules. Mitochondrial protection can be achieved during AKI using a mitoprotective agent like SS-31, fibroblast growth factor (FGF2), or compounds that activate the mitochondrial ATP-sensitive potassium channel. Additionally, the Drp1 inhibitor P110 can alleviate aberrant mitochondrial fragmentation and AKI by suppressing the interaction between Drp1 and Fis1 [275]. Replacing damaged mitochondria aids in restoring mitochondrial homeostasis [276, 277]. Despite the promising potential, further research, including well-designed clinical trials, is critically needed. Nonetheless, mitochondrial replacement therapy undoubtedly provides a new and potentially revolutionary therapeutic approach for the management of AKI. Shen Z created a CD44-targeted and ROS-responsive CS-BR-mediated multifunctional liposome loading celastrol (CS-BR@CLT) for the targeted therapy of AKI, which exhibited multiple anti-AKI functions [278]. Its usage significantly ameliorates AKI attributable to ischemia–reperfusion and helps to preserve renal function by inhibiting apoptosis, protecting mitochondria, promoting autophagy, regulating macrophage polarization, and alleviating interstitial inflammation. Inhibition of CXCL5 may mitigate acute tubular injury and avert the subsequent progression from AKI to CKD [279].

Besides, targeting ER stress-related treatment mesencephalic astrocyte-derived neurotrophic factor (MANF) inhibited JNK/TLR4/NF-κB signaling [280]. WIP1 inhibitor (CCT007093) functions as a positive regulator of renal tubular pyroptosis, primarily mediated by phospho-p38 MAPK in sepsis-associated AKI [281]. Vaccines are anticipated to emerge as a groundbreaking approach for the prophylaxis and management of AKI. Pre-infusion of tolerogenic CD11c^+^DCs holds the potential to effectively replace the regulatory function of damaged intracellular pathways in cells (DIPC) on DCs and T-cells, thereby alleviating I/R-AKI [282]. The emergence of nanomedicine paved the way for improving AKI. The H_2_S-generating pre-nanozyme Pt_5.65_S demonstrates inducible antioxidant enzyme‐like activity within an acidic and inflammatory microenvironment. It also demonstrates protective effects against damage caused by ROS and reactive nitrogen species (RNS), even at extremely reduced doses, which significantly improved treatment outcomes in mouse models of kidney ischemia–reperfusion injury and cisplatin-induced AKI [283]. Managing ROS and inflammation is emerging as a potential strategy for mitigating AKI. Polyethylene glycol (PEG)-coated osmium nanozymes (Os) have shown promise in this regard. When combined with photothermal therapy (PTT) and chemotherapy, these nanozymes effectively regulate inflammation by scavenging ROS and generating oxygen, thereby alleviating cisplatin-induced AKI [284]. The nanoplatform offers provides a new therapeutic approach for addressing AKI caused by rhabdomyolysis. MPD NPs effectively rescued renal function in vivo by targeting ferroptosis to downregulate the antioxidant pathway, thereby mitigating oxidative stress and inflammatory responses and inhibiting renal tubular cell apoptosis [285]. Targeting ferroptosis with drugs offers a new and highly promising therapeutic avenue for the treatment of kidney diseases. Klotho, an anti-aging protein predominantly synthesized by TECs, is regarded as a potential anti-inflammatory molecule in AKI. Nanoparticle-mediated Klotho gene therapy for preventing the transition from AKI to CKD [286]. New therapeutic options are currently being investigated for different types of AKI. For sepsis-associated AKI, the potential benefits of alkaline phosphatase and L-carnitine are being explored. In hospitalized patients with AKI, the role of vitamin D is under study. Additionally, p53-targeted short-interfering RNA is being examined as a possible treatment for AKI following cardiac surgery.

### Renal replacement therapy (dialysis)

Renal replacement therapy (RRT) is the primary treatment modality for AKI, particularly in cases associated with sepsis. Intermittent hemodialysis (IHD) and continuous RRT (CRRT) are the two main forms of RRT used to treat patients with severe AKI. The cornerstone of managing early AKI lies in preventive strategies, such as optimizing volume hemodynamics and status, as well as avoiding nephrotoxins. However, for severe AKI, RRT remains the sole therapeutic option. The optimal timing for initiating RRT has long been a subject of debate. Traditionally, RRT is initiated for the acute management of critical complications arising from AKI. The choice of the most suitable RRT option for critically ill patients with AKI remains a contentious issue. A secondary analysis of the Standard versus Accelerated Renal Replacement Therapy in AKI (STARRT-AKI) trial unveiled that among critically ill patients suffering from severe AKI, the commencement of CRRT was linked to a substantial decrease in the combined outcome of death or dependence on RRT at 90 days, compared to IHD [287]. Another secondary analysis of two multicenter randomized controlled trials, namely the AKIKI and IDEAL-ICU studies, indicated that using CRRT as the first modality did not appear to confer any benefits regarding survival or renal function restoration. In fact, it might have correlated with less positive outcomes in patients with less advanced disease when compared to IHD [288]. Unexpectedly, among critically ill patients with AKI, an expedited renal-replacement approach did not result in a decreased likelihood of death within 90 days compared to a standard strategy (STARRT-AKI ClinicalTrials.gov number, NCT02568722) [289]. The Worldwide Exploration of Renal Replacement Outcomes Collaborative in Kidney Disease (WE-ROCK) on a contemporary cohort of children weighing ≤ 10 kg treated with continuous kidney replacement therapy (CKRT) for AKI and/or fluid overload. ICU mortality rates are reduced when compared to those in prospective pediatric CRRT. The reasons for initiating treatment early are manifold. It can help to prevent severe electrolyte and acid–base disturbances, achieve better volume control, and avert uremic complications as well as rapid mortality due to the adverse effects of renal failure. Moreover, when determining whether to commence RRT, it is crucial to consider the trends in laboratory test results rather than relying solely on the creatinine threshold or blood urea nitrogen (BUN) level. However, starting RRT before it is truly necessary may expose patients to a variety of negative consequences. Surveillance and prevention of fluid overload during extracorporeal membrane oxygenation (ECMO) and CKRT are essential to reduce the risk of mortality [290]. The application of the oXiris filter in CRRT has been linked to diminished inflammatory damage and enhanced renal perfusion. However, in cases of sepsis-associated AKI, it does not appear to correlate with enhanced 28—day recovery of renal function or reduced 28-day all-cause mortality [291]. In a retrospective study conducted at a single center, low-effluent volume CRRT-PIRRT demonstrated comparable outcomes to standard effluent volume CRRT-PIRRT, aligning with the findings of previous observational research [292]. Artificial intelligence (AI) by establishing longitudinal, multimodal models improved early detection of AKI and prediction of CRRT requirement, facilitating timely interventions and thereby optimizing AKI management [293]. Despite the diverse ethical, climatic, geographic, and socioeconomic contexts in Asia that lead to the utilization of various RRT modalities, it is of paramount importance to ensure optimal care for all AKI patients.

## Conclusions and perspectives

Recent advances in understanding the pathophysiology, risk factors, and causes of AKI highlight its significance in AKI genesis and development. Among them, onco-nephrology is an emerging and rapidly evolving subspecialty that delves into the intricate and multifaceted relationships between the kidneys and cancer. Prior research has elucidated the “two-hit” mechanism explaining the link between AKI and clinical conditions. For example, cancer acts as the initial insult or “first hit” to the kidney, with subsequent exposure to diverse treatments exacerbating toxicity and serving as the subsequent insult or “second hit” [294]. These discoveries underscore the critical interplay between remote malignancies and the kidney, with direct clinical relevance. Furthermore, they provide fresh perspectives on AKI management and treatment. Nonetheless, it is essential to recognize specific limitations. The complexity and dynamic nature of human tumors were inadequately captured in the animal model used. The traditional cisplatin-induced AKI mouse model lacks tumor presence. To enhance the relevance to clinical scenarios for this subgroup, it is essential to develop more comprehensive animal models that incorporate factors like appropriate dosing regimens of cisplatin, age, gender, genetic background, and tumor grafts, thus increasing model reproducibility and translatability significantly. Additionally, variations in AKI assessment criteria among previous studies may lead to inconsistencies in AKI evaluations.

Although AKI was well-defined and studied, it was clearly different from CKD. Nevertheless, there is a substantial deficiency in research, care, and guidance for patients who have abnormalities in kidney function and/or structure but do not meet the diagnostic criteria for either AKI or CKD. AKD is addressed and defined by functional or structural kidney abnormalities that have health implications and last for a duration of ≤ 3 months. AKD encompasses AKI, but more significantly, it also involves kidney functional abnormalities that are less severe than AKI or that evolve over a period exceeding 7 days [295]. Hence, future exploration of AKD is of importance. Timely detection and treatment of AKI are crucial to prevent its progression to AKD and mitigate long-term adverse outcomes [296]. The intensity and duration of early postoperative AKI are linked to a higher incidence of AKD, and early postoperative AKI has a strong association with AKD [297]. Pseudo-AKI occurrences pose challenges in clinical practices, particularly concerning the impact of diverse targeted agents, highlighted in recent literature [298–307] (Table 5). Notably, some tyrosine kinase inhibitors (e.g., sunitinib, sorafenib) and BCR-ABL inhibitors (e.g., dasatinib, nilotinib) have demonstrated potential for inducing pseudo-AKI in vitro, although clinical verification is lacking [301, 308]. Lastly, subclinical AKI may manifest during cancer treatments, presenting symptoms like hypertension from renin-angiotensin axis activation, flank pain due to renal capsule stretching, and hematuria.

**Table 5:** The mechanism of Targeted therapy-associated pseudo-AKI

Targeted therapy	Drugs	Targeted transporters	Mechanism	References
ALK inhibitor	crizotinib	-	eGFR is reduced	[299]
	crizotinib	MATE1, OCT2	creatinine uptake	[300]
	ceritinib	OCT2	-	[301]
	lorlatinib	MATE1	-	[301]
	brigatinib	MATE1, MATE2k	-	[301]
	Lorlatinib	MATE1	-	[301]
	entrtinib	MATE1	-	[301]
MET inhibitor	topotinib	OCT2, MATE1, MATE2k	inhibit creatinine tubular secretion, falsely low GFR	[302]
	capmatinib	MATE1, MATE2k	Inhibit creatinine tubular secretion, falsely low GFR	[303]
BCR-ABL inhibitor	imatinib	OCT2, MATE1	inhibit creatinine uptake	[304]
		OCT1, OCT3, MATE1, MATE2k	inhibit metformin uptake	[304]
CKD4/6 inhibitors	abemaciclib	OAT2, MATE1, MATE2-K	inhibit renal tubular secretion without changing glomerular filtration rate	[305]
	palbociclib	OCT2	-	[306]
HER2 inhibitor	Tucatinib	OCT2, MATE1	tubular secretion of creatinine, mild SCr increases that do not signify kidney impairment	[307]
PARP inhibitor	olaparib	MDR1, OCT1, OCT2, OATP1B1, OAT3, MATE1 and MATE2K	-	[308]

*TKIs* Tyrosine kinase inhibitors, *AKI* Acute kidney injury, *MATE 1/2 K* multidrug and toxin extrusion protein 1 and 2 K, *MRPs* Multidrug resistance-associated protein, *ATP7A* P-type copper transporting ATPases, *DDR* DNA damage response or repair, *eGFR* Estimating Glomerular Filtration Rate, *OCT1/2/3* Organic cation transporter 1/2/3, *OATPs* organic anion transporting polypeptides, *OAT3* organic anion transporter 3, *ALK* anaplastic lymphoma kinase, *MET* mesenchymal-epithelial transition, *CDK4/6* Cyclin-dependent kinase 4/6 inhibitor, *HER2* human epidermal growth factor receptor 2; *PARP* Poly ADP Ribose Polymerase, *SCr* serum creatinine

To date, a group of studies revealed that it’s essential to screen and utilize biomarkers in early cancer detection and pinpointing the highest-risk patients [309]. Currently, novel tumor markers have emerged, leveraging genetic mutations or epigenetic modifications frequently observed in cancer, including abnormal DNA methylation, exosomes, metabolites, mRNAs, or cell-free DNA (cfDNA), with urinary markers also being explored [310]. Therefore, a thorough comprehension of the fundamental mechanisms of cancer-associated AKI is crucial to advocate AKI-related biomarker screening for early detection and subsequent protective measures to maintain renal function during cancer progression and exposure to diverse therapies. While biomarkers for predicting AKI and small molecular inhibitors for AKI alleviation have emerged, it is vital to tackle the challenges linked to identifying AKI or subclinical AKI within the cancer context. Currently, only a limited number of novel biomarkers have been incorporated into conventional clinical management. Shi K et al. performed a meta-analysis and unveiled that CCL14 emerges as the optimal biomarker for predicting persistent AKI, particularly persistent stage 2–3 AKI [311]. With the advent of artificial intelligence (AI), its application has predominantly been centered on predicting AKI. However, more advanced methods are also being employed to categorize existing AKI into distinct phenotypes [312]. Early prediction of AKI by novel biomarkers or AI-based prediction models may provide a crucial opportunity for AKI prevention [313]. Postoperative acute kidney injury requiring dialysis (PO-AKID) is a serious adverse event strongly associated with acute morbidity and mortality, as well as long-term prognosis. A web-based risk calculator called the PO-AKID-teller can accurately predict an individual's risk for PO-AKID in an easy-to-understand way. This tool may help with informed decision-making, patient counseling, perioperative optimization, and longer-term care planning [314].

Timely intervention is critical for controlling AKI. Targeted therapies should be devised, focusing on the pivotal molecular pathways implicated in AKI pathogenesis. Uncertainty persists regarding the association between AKI and novel agents like CDK4/6 inhibitors, necessitating extensive prospective studies to elucidate incidence rates and risk factors for AKI in patients undergoing such therapies [315]. Likely, some innovative cancer treatments regarding small renal mass such as focal therapy and active surveillance and associated renal function detriments including AKI are arrived and paid more focus [316]. As for metastatic renal carcinoma, cytoreductive nephrectomy and metastasectomy showed different advantages [317]. Therapeutic targeting of mitochondria is an emerging strategy for AKI. Yet, additional studies are required prior to its application in clinical therapeutic contexts. Meanwhile, postponing the progression from AKI to CKD is a viable and manageable treatment approach. Specifically, Zn-D/DTH has been shown to reduce renal IRI and delay the transition from AKI to CKD by downregulating the TLR4/MyD88/NF-κB signaling cascade and reducing the expression of apoptotic caspases, thereby inhibiting inflammation and decreasing cell apoptosis [318]. Furthermore, recognizing high-risk individuals and clinical conditions, as well as high-risk situations and procedures, is an important step toward preventing AKI. Early detection of AKI may present a critical opportunity for AKI prophylaxis. Once considered a transient condition, AKI is now recognized as having significant long-term consequences. Acute alterations in kidney function are increasingly understood to be associated with the progression to CKD, adverse cardiovascular effects, persistent functional impairment, and increased mortality. The majority of significant “treatments” for AKI majority address complications of AKI rather than AKI itself. On the same note, AKI causes mutual damage within multi-organ injuries. During cardiac arrest, ischemia–reperfusion followed by successful cardiopulmonary resuscitation was prone to lead to AKI. Traditional complications of AKI, including electrolyte imbalances, uremia, and fluid overload, have long been identified as major factors contributing to adverse pulmonary outcomes in patients with AKI, respiratory complications/inflammatory lung injury. Moreover, AKI also led to intestinal injury, liver injury, and immunoparalysis. Likewise, the injuries of these organs also resulted in or exacerbated AKI. Most of these interventions are also highly debated. Among them, diuretics and bicarbonate are particularly contentious. Regarding diuretics, KDIGO advises against their use for treating AKI, except in cases of fluid overload. As for bicarbonate supplementation, the evidence from randomized controlled trials is extremely limited, which makes its efficacy highly questionable. It is crucial to establish a sustainable infrastructure that can provide appropriate care for all AKI patients.

In summary, AKI remains a catastrophic issue among hospitalized patients, particularly for those in critical condition. Although the development of biomarkers for early diagnosis is still ongoing, raising awareness of AKI is crucial for early detection. This early recognition is highly contingent upon the specific context and the resources available at the site where AKI occurs. These findings can provide the pathological mechanism of AKI and may establish novel therapeutic strategies. Undoubtedly, future investigations will identify additional biomarkers, targets, and therapies for AKI.

## Supplementary Information

Supplementary Material 1: Table 1. The patients-related risk factor associated with AKI. Table 2. AKI-related anti-cancer drugs

## Data Availability

Not applicable.
