# Supplementary material for: Acute kidney injury: pathogenesis and therapeutic interventions
Source: Mol Biomed. 2025 Sep 5;6:61. doi: 10.1186/s43556-025-00293-4 (PMC12413393; doi:10.1186/s43556-025-00293-4)
Supplement: Supplementary file 1 — Supplementary Material 1: Table 1. The patients-related risk factor associated with AKI. Table 2. AKI-related anti-cancer drugs [file 43556_2025_293_MOESM1_ESM.docx]

**Acute Kidney Injury：pathogenesis and therapeutic interventions**

Xiaoqin Xu PhD^⁋ 1,2^, Tingting Zeng PhD^⁋ 1^, Si Chen MM^1^, Na Tian MM^3^, Chunying Zhang PhD^1^, Yuemei Chen BS^1^, Shanying Deng BS^1^, Zhigang Mao MM^1^, Juan Liao BS^1^, Tonghao Zhang PhD^4^, Yi He PhD^5^, Wei Wang PhD^5^, Pan Chen PhD*^6^, Yali Song PhD*^1^

^1^ Department of Laboratory Medicine, Clinical Laboratory Medicine Research Center, West China Hospital, Sichuan University, Sichuan Clinical Research Center for Laboratory Medicine, Chengdu, 610041, Sichuan Province, People’s Republic of China

^2^ Department of Clinical Laboratory, Shanxi Province Cancer Hospital, Shanxi Hospital Affiliated to Cancer Hospital, Chinese Academy of Medical Sciences, Cancer Hospital Affiliated to Shanxi Medical University, Taiyuan, 030013, Shanxi Province, People’s Republic of China

^3^ Department of Anesthesiology, Qingdao Eighth People’s Hospital, Qingdao, 266000, Shandong Province, People’s Republic of China

^4^ Department of Statistics, University of Virginia, Charlottesville, 22903, USA

^5^ Gastroenterology and Urology Department Ⅱ, Hunan Cancer Hospital/the Affiliated Cancer Hospital of Xiangya School of Medicine, Central South University; Clinical Research Center For Gastrointestinal Cancer In Hunan Province, Changsha, 410013, Hunan Province, People’s Republic of China

^6^ Hunan Cancer Hospital and the Affiliated Cancer Hospital of Xiangya School of Medicine, Central South University, Changsha, 410013, Hunan Province, People’s Republic of China

***Corresponding author:**

Yali Song, E-mail: songyl@scu.edu.cn

Pan Chen, E-mail: chenpan@hnca.org.cn

^⁋^ These authors contributed equally to this work.

**Funding information**: This study was supported in part by National Natural Science Foundation of China (No. 82302632, Yali Song), Natural Science Foundation of Sichuan Province (No. 2025ZNSFSC0550, Yali Song) and the grants from the Clinical Research Center for Gastrointestinal Cancer in Hunan Province (No. 2021SK4016, Yi He and Wei Wang).

**Supplementary** **Table 1 The patients-related risk factor associated with AKI**

| **Risk factors** | **Type of cancer** | **Conditions** | ***OR*** | ***95%CI*** | ***P*** | **References** | |
| --- | --- | --- | --- | --- | --- | --- | --- |
| **Demographic characteristic** |  |  |  |  |  |  |  |
| Male | - | HA | 1.07 | 1.02-1.12 | 0.008 | (8) |  |
| Male sex | Esophageal cancer | pre-surgery | 1.77 | 1.10-2.81 | 0.015 | (12) |  |
| Male | - | - | 1.15 | 1.14-1.16 | < 0.001 | (23) |  |
| Female sex | RCC | - | 0.64 | 0.59–0.69 | < 0.001 | (15) |  |
| Female | Lung cancer | - | 0.71 | 0.585-0.854 | <0.001 | (36) |  |
| Age | Esophageal cancer | pre-surgery | 1.02 | 1.00-1.04 | 0.027 | (12) |  |
| Age 60-69 (ys) | RCC | - | 1.51 | 1.35–1.68 | < 0.001 | (15) |  |
| Age 70-79 (ys) | RCC | - | 1.80 | 1.58–2.06 | < 0.001 | (15) |  |
| Age ≥80 (ys) | RCC | - | 2.35 | 1.99–2.77 | < 0.001 | (15) |  |
| Older age | - | - | 1.01 | 1.00-1.03 | 0.039 | (22) |  |
| Age | Lung cancer | - | 1.02 | 1.007-1.024 | <0.001 | (36) |  |
| Black race | RCC | - | 1.85 | 1.59–2.14 | < 0.001 | (15) |  |
| Smoking history | Esophageal cancer | - | 3.029 | 1.092-8.399 | 0.033 | (11) |  |
| BMI | Esophageal cancer | pre-surgery | 1.10 | 1.07-1.14 | < 0.001 | (12) |  |
| BMI | Colorectal cancer | - | 1.04 | 1.00–1.09 | 0.035 | (13) |  |
| Advanced cancer stage | - | - | 1.41 | 1.28-1.54 | <0.001 | (32) |  |
| **Baseline levels of serum creatinine or eGFR** |  |  |  |  |  |  |  |
| Preoperative serum creatinine level | Esophageal cancer | - | 1.040 | 1.012-1.069 | 0.005 | (11) |  |
| eGFR<60 mL/min/1.73 m^2^ | advanced ovarian cancer | - | 2.704 | 1.373-5.322 | 0.004 | (16) |  |
| Baseline eGFR | head and neck cancer | - | 1.03 | 1.0-1.1 | 0.04 | (19) |  |
| Initial eGFR < 60mL/min/1.73 m^2^ | Lung cancer | - | 2.03 | 1.557-2.655 | <0.001 | (36) |  |
| **Comorbidities** |  |  |  |  |  |  |  |
| CKD | - | - | 1.80 | 1.67-1.93 | <0.001 | (32) |  |
| Preoperative CKD stage | RCC | - | 12.52 | 10.62-14.77 | < 0.001 | (15) |  |
| Preexisting CKD | - | - | 2.90 | 1.65-5.11 | < 0.001 | (22) |  |
| Chronic renal disease | - | - | 6.8 | 1.7-80.1 | 0.017 | (35) |  |
| DM | - | - | 1.43 | 1.37-1.50 | <0.001 | (32) |  |
| DM | Colorectal cancer | - | 1.58 | 1.18–2.13 | 0.002 | (13) |  |
| DM | Lung cancer | - | 1.53 | 1.220-1.924 | <0.001 | (36) |  |
| Shock | - | CA | 7.07 | 5.47-9.08 | <0.001 | (8) |  |
| Shock | - | HA | 3.18 | 2.82-3.58 | <0.001 | (8) |  |
| Urinary infection | - | CA | 2.56 | 1.90-3.41 | <0.001 | (8) |  |
| Urinary tract obstruction | - | CA | 6.11 | 4.82-7.68 | <0.001 | (8) |  |
| Urinary tract obstruction | - | HA | 1.94 | 1.60-2.35 | <0.001 | (8) |  |
| Respiratory failure | - | CA | 2.44 | 1.88-3.14 | <0.001 | (8) |  |
| Respiratory failure | - | HA | 1.85 | 1.63-2.09 | <0.001 | (8) |  |
| Sepsis | - | CA | 2.70 | 1.86-3.82 | <0.001 | (8) |  |
| Sepsis | - | HA | 1.50 | 1.29-1.75 | <0.001 | (8) |  |
| Sepsis | - | - | 4.4 | 1.9-10.1 | <0.001 | (35) |  |
| Gastrointestinal bleeding | - | CA | 2.33 | 1.77-3.03 | <0.001 | (8) |  |
| Gastrointestinal bleeding | - | HA | 1.46 | 1.28-1.68 | <0.001 | (8) |  |
| Myocardial infarction | - | CA | 2.03 | 1.03-3.73 | 0.03 | (8) |  |
| Myocardial infarction | - | HA | 1.46 | 1.10-1.95 | 0.010 | (8) |  |
| Heart failure | - | CA | 2.12 | 1.66-2.69 | <0.001 | (8) |  |
| Heart failure | - | HA | 1.33 | 1.17-1.53 | <0.001 | (8) |  |
| Cardiovascular disease | RCC | - | 1.61 | 1.48–1.75 | < 0.001 | (15) |  |
| Congestive heart failure | - | - | 17.1 | 1.7-80.1 | 0.015 | (35) |  |
| Lung infection | - | CA | 2.63 | 2.30-3.00 | <0.001 | (8) |  |
| Lung infection | - | HA | 1.23 | 1.14-1.32 | <0.001 | (8) |  |
| Pneumonia | Esophageal cancer | post-surgery |  |  | 0.005 | (12) |  |
| Liver disease | - | HA | 1.42 | 1.32-1.53 | <0.001 | (8) |  |
| Hypertension | - | HA | 1.11 | 1.04-1.18 | 0.001 | (8) |  |
| Hypertension | Esophageal cancer | - | 6.422 | 2.736-15.070 | <0.001 | (11) |  |
| Hypertension | Colorectal cancer | - | 1.40 | 1.06–1.84 | 0.016 | (13) |  |
| Hypertension | RCC | - | 0.64 | 0.59–0.71 | < 0.001 | (15) |  |
| Hypertension | Head and neck cancer | - | 3.26 | 1.4-7.6 | 0.005 | (19) |  |
| Hypertension | - | - | 2.30 | 1.41-3.76 | < 0.001 | (23) |  |
| Dyslipidemia | Esophageal cancer | pre-surgery | 2.14 | 1.34-3.44 | 0.002 | (12) |  |
| Atrial fibrillation | Esophageal cancer | post-surgery |  |  | 0.013 | (12) |  |
| Albumin | Colorectal cancer | - | 0.57 | 0.44–0.74 | < 0.001 | (13) |  |
| Albumin | Lung cancer | - | 0.65 | 0.533-0.783 | <0.001 | (36) |  |
| serum albumin level < 3.8 g/dl | Brain tumor | - | 1.981 | 1.02-3.84 | 0.043 | (25) |  |
| Obesity | RCC | - | 1.51 | 1.33–1.71 | < 0.001 | (15) |  |
| Anemia | RCC | - | 2.28 | 1.89–2.74 | < 0.001 | (15) |  |
| Anemia | Lung cancer | - | 1.38 | 1.145-1.660 | 0.001 | (36) |  |
| Hypercalcemia | - | - | 8.4 | 1.3-46.1 | 0.028 | (35) |  |
| Extrarenal irAEs | - | - | 2.73 | 1.77-4.22 | < 0.001 | (23) |  |
| **Concomitant agents** |  |  |  |  |  |  |  |
| ACEI | - | Age ≥ 66(ys) | 1.30 | 1.23-1.38 | <0.001 | (32) |  |
| ACEI or ARB | Advanced ovarian cancer | - | 3.122 | 1.545-14.892 | 0.039 | (16) |  |
| ACEI/ARB | - | - | 1.76 | 1.15-2.68 | 0.009 | (22) |  |
| Beta-blocker | - | Age ≥ 66(ys) | 1.10 | 1.04-1.17 | <0.001 | (32) |  |
| Calcium channel blocker | - |  | 1.18 | 1.07-1.30 | 0.001 | (32) |  |
| Diuretic | - | Age ≥ 66(ys) | 1.20 | 1.14-1.28 | <0.001 | (32) |  |
| Diuretics | - | - | 1.78 | 1.32-2.40 | <0.001 | (22) |  |
| Diuretic | - | - | 1.77 | 1.20-2.61 | 0.004 | (23) |  |
| RAASi treatment | Head and neck cancer | - | 0.13 | 0.033-0.44 | 0.002 | (19) |  |
| PPI | - | - | 2.23 | 1.88-2.64 | <0.001 | (22) |  |
| PPI | - | - | 2.07 | 1.58-2.71 | < 0.001 | (23) |  |
| NSAID | - | - | 2.61 | 1.90-3.57 | <0.001 | (22) |  |
| NSAIDs | - | - | 1.84 | 1.04-3.24 | 0.04 | (23) |  |
| Fluindione | - | - | 6.48 | 2.72-15.46 | <0.001 | (22) |  |
| **Therapy** |  |  |  |  |  |  |  |
| Systemic therapy | - | 90 days | 2.34 | 2.24-2.45 | <0.001 | (32) |  |
| Duration of surgery | Esophageal cancer | - | 1.009 | 1.005-1.014 | <0.001 | (11) |  |
| Radical nephrectomy | RCC | - | 1.21 | 1.07–1.36 | 0.003 | (15) |  |
| Higher cisplatin dose ≥70 mg/m^2^ | Advanced ovarian cancer | - | 3.668 | 1.336-10.070 | 0.012 | (16) |  |
| Number of chemotherapy cycles | Head and neck cancer | - | 1.82 | 1.2-2.9 | 0.007 | (19) |  |
| ICIs | - | - | 2.45 | 1.40-4.31 |  | (22) |  |
| Treatment with CTLA-4 | - | - | 1.33 | 1.08-1.64 | 0.007 | (23) |  |
| ICU admission | - | - | 5.8 | 2.1-16.2 | 0.001 | (35) |  |

AKI: Acute kidney injury; CA: Community-acquired, HA: Hospital-acquired, CKD: chronic kidney disease, ACEI: Angiotensin-converting enzyme inhibitors; ARB: angiotensin receptor blockers; PPI: proton pump inhibitor; NSAID: Nonsteroidal anti-inflammatory drug; DM: Diabetes mellitus; BMI: Body mass index; eGFR: Estimating Glomerular Filtration Rate; irAEs: Immune-related adverse events; CTLA-4: Cytotoxic T Lymphocyte antigen 4; ICIs: Immune checkpoint inhibitors; ICU: Intensive care unit; RT: radiotherapy; ADT: Androgen deprivation therapy; RCC: Renal cell carcinoma

**Supplementary Table 2 AKI-related anti-cancer drugs**

| Therapeutic methods | Drugs |
| --- | --- |
| **Chemotherapy drugs** | cisplatin, carboplatin, oxaliplatin, methotrexate, sulfadiazine, sulfamethoxazole, ndinavir, atazanavir, darunavir |
| **Immunotherapy drugs** |  |
| CTLA-4 antibodies | ipilimumab, tremelimumab |
| PD-1 antibodies | nivolumab, pembrolizumab |
| PD-L1 antibodies | atezolizumab |
| **Targeted drugs** |  |
| *Proteasome inhibitor* | carfilzomib |
| TKIs | vandetanib |
| EGFR inhibitors | erlotinib, gefitinib, afatinib |
| monoclonal antibodies | cetuximab, panitumumab |
| Anti-EGFR | trametinib and dabrafenib |
| VEGF inhibitors | bevacizumab, aflibercept |
| BRAF inhibitors | vemurafenib and dabrafenib |
| ALK-1 inhibitors | crizotinib, alectinib |

Note: CTLA-4: Cytotoxic T Lymphocyte antigen 4; PD-1: Programmed cell death Protein 1; PD-L1: Programmed death-ligand 1; TKIs: Tyrosine kinase inhibitors; EGFR: Epidermal Growth Factor Receptor; BRAF: v-Raf murine sarcoma viral oncogene homolog B ; VEGF: Vascular endothelial growth factor; ALK: Anaplastic lymphoma kinase
